# HtrA2/Omi Terminates Cytomegalovirus Infection and Is Controlled by the Viral Mitochondrial Inhibitor of Apoptosis (vMIA)

**DOI:** 10.1371/journal.ppat.1000063

**Published:** 2008-05-09

**Authors:** A. Louise McCormick, Linda Roback, Edward S. Mocarski

**Affiliations:** Department of Microbiology and Immunology and Emory Vaccine Center, Emory University School of Medicine, Atlanta, Georgia, United States of America; Washington University School of Medicine, United States of America

## Abstract

Viruses encode suppressors of cell death to block intrinsic and extrinsic host-initiated death pathways that reduce viral yield as well as control the termination of infection. Cytomegalovirus (CMV) infection terminates by a caspase-independent cell fragmentation process after an extended period of continuous virus production. The viral mitochondria-localized inhibitor of apoptosis (vMIA; a product of the UL37x1 gene) controls this fragmentation process. UL37x1 mutant virus-infected cells fragment three to four days earlier than cells infected with wt virus. Here, we demonstrate that infected cell death is dependent on serine proteases. We identify mitochondrial serine protease HtrA2/Omi as the initiator of this caspase-independent death pathway. Infected fibroblasts develop susceptibility to death as levels of mitochondria-resident HtrA2/Omi protease increase. Cell death is suppressed by the serine protease inhibitor TLCK as well as by the HtrA2-specific inhibitor UCF-101. Experimental overexpression of HtrA2/Omi, but not a catalytic site mutant of the enzyme, sensitizes infected cells to death that can be blocked by vMIA or protease inhibitors. Uninfected cells are completely resistant to HtrA2/Omi induced death. Thus, in addition to suppression of apoptosis and autophagy, vMIA naturally controls a novel serine protease-dependent CMV-infected cell-specific programmed cell death (cmvPCD) pathway that terminates the CMV replication cycle.

## Author Summary

Cellular suicide is an effective host defense mechanism to control viral infection. Host cells encode proteins that induce infected cell death while viruses encode proteins that prevent death and facilitate viral replication. Human cytomegalovirus encodes vMIA to suppress host-initiated death pathways. Cytomegalovirus infection is controlled by the evolutionarily ancient mitochondrial serine protease, HtrA2/Omi. HtrA2/Omi levels rise dramatically within mitochondria at late times during viral infection, eventually overcoming viral control of a cell death pathway that is dependent on this serine protease and independent of the well-studied apoptotic cell death pathway that conventionally depends upon a class of proteases called caspases. vMIA naturally counteracts HtrA2/Omi-dependent cell death and allows infected cells to survive and produce virus for several days. The natural inhibitory role of vMIA can be overwhelmed by overexpression of HtrA2/Omi in virus-infected cells, but uninfected cells are insensitive to HtrA2/Omi-induced death. The broad distribution of HtrA2/Omi within mammalian host species suggests this may represent an ancient antiviral response or a process of viral *detente* that establishes the timing of infection. Either way, the success of cytomegalovirus rests in the balance between cell death initiation and the viral cell death suppressor vMIA.

## Introduction

Cell death is central to viral infection, as an evolutionarily-conserved means to eliminate intracellular pathogens and as a way that lytic viruses mediate release of progeny. Human cytomegalovirus (CMV), the major infectious cause of birth defects as well as an important cause of opportunistic disease worldwide [1], remains cell-associated during productive replication. Release of progeny virus depends upon the exocytic pathway [1] and continues until cells die via a poorly understood fragmentation process. CMV is well-armed to modulate cell-intrinsic as well as extrinsic innate and adaptive host clearance pathways [1]. The product of the UL37x1 gene, vMIA, a potent suppressor of apoptosis [2]–[4], also controls the timing of infected cell death [5]–[7]. Premature death in vMIA-mutant virus-infected cells reduces the period of progeny release by three to four days [5]–[7] without affecting cell-to-cell spread [5]. All vMIA-mutant viruses exhibit this premature death phenotype, but the involvement of caspases and the impact on viral yield varies with CMV strain. AD169*var*ATCC strain (AD-BAC) depends upon vMIA to a greater extent [6],[7] than Towne*var*ATCC (Towne-BAC), although vMIA prolongs the period of viral replication and release in both strains [5]. Importantly, vMIA from either strain retains the capacity to block caspase-dependent apoptosis [5]. The caspase-independent death pathway that is blocked by vMIA is not known. Other cell death suppressors are encoded by CMV [1], but, aside from vMIA, only UL38 has been implicated in control of infected fibroblast death to prolong replication [8],[9]. Studies to date reveal a complexity of infected cell death and a need for a more complete understanding of events that naturally terminate CMV infection.

Major pathways associated with death (apoptosis, necrosis, and autophagy) are triggered by specific host cell and immune system initiators and exhibit characteristic molecular events and cell morphological changes [10]–[12]. Proteases in the caspase, calpain, lysosomal cathepsin, and proteasomal serine protease classes are central to the execution of various death pathways. The fact that the premature death induced by vMIA-mutant CMV is resistant to inhibitors of caspases, cathepsins, and calpains [5] suggests a novel programmed pathway distinct from characterized death pathways [11],[13]. For those viral strains that have been characterized, infected cell death initiates approximately 7 to 10 days after infection of fibroblasts. In contrast, the premature death that occurs in vMIA mutant virus infection initiates 3 to 4 days postinfection [5]–[7]. The 12–24 h timing of individual cell fragmentation, association with cytopathic effect (CPE), and nominal impact of vMIA [5] all suggest that the final stages of productive replication terminate with a CMV infected cell-specific programmed cell death (cmvPCD).

Many DNA viruses encode antiapoptotic functions that sustain replication in the face of cell-intrinsic defenses [14]–[16]. vMIA equips CMV to counteract intrinsic host clearance pathways leading to cell death [5]–[7]. As an outer mitochondrial membrane protein, vMIA sits in a central position analogous to antiapoptotic Bcl-2 family members Bcl-2 and Bcl-x_L_ [2], and prevents the formation of a mitochondrial permeability transition pore, release of cytochrome *c* and proapoptotic factors into the cytoplasm, and activation of executioner caspases. Unlike antiapoptotic Bcl-2 family members [2],[5],[17], vMIA lacks Bcl-2 homology domains but depends on an antiapoptotic domain that mediates interaction with GADD45 family members [18],[19]. vMIA also recruits BAX to mitochondria [20],[21] and disrupts mitochondrial networks [22]. This disruption normally accompanies BAX oligomerization at the outer mitochondrial membrane [23],[24], although vMIA mutants that fail to bind BAX continue to disrupt networks [25]. The vMIA-dependent recruitment of BAX does not lead to the formation of a transition pore complex or release of proapoptotic mediators [20],[21]. The contribution of BAX oligomerization or mitochondrial network disruption to cell death suppression remains to be investigated. Although both of these events are signs of apoptosis [23],[24],[26],[27] neither mitochondrial network disruption [28] nor BAX oligomerization [29],[30] are sufficient to induce apoptosis. These alterations are also associated with vMIA-mediated suppression of cell death during viral infection where the pathway(s) of death are not fully understood.

Consequences of mitochondrial release of proapoptotic mediators have been extensively studied [10], [31]–[33]. Cytochrome *c* controls apoptosome formation and downstream executioner caspase activation. Endonuclease G and apoptosis-inducing factor (AIF) promote nuclear events. Mitochondrial release of Smac/DIABLO and HtrA2/Omi overcomes the activity of inhibitor of apoptosis proteins (IAPs). The HtrA2/Omi proenzyme is processed within the mitochondria, removing a mitochondrial targeting sequence (amino terminal 33 amino acids) and a transmembrane domain [34]–[36]. Mature, active HtrA2/Omi resides in the intermembrane space, and is released into the cytoplasm through the transition pore complex at the same time as cytochrome *c*. Release of the serine protease HtrA2/Omi from mitochondria can result in two downstream effects: (1) cleavage of IAPs and an ultimate increase in caspase-dependent death and (2) trigger IAP-independent, caspase-independent death [37]. This latter pathway is also induced by extramitochondrial overexpression of HtrA2/Omi in the cytoplasm [35], [37]–[40]. The role of this serine protease as an inducer of cell death [38],[39] in mammalians seems opposite the role of the founding member of this protein family as a pro-survival serine protease in eubacteria [41]–[43]. Here, we demonstrate the central role of HtrA2/Omi executing a serine protease-dependent pathway that is controlled by vMIA during infection.

## Results

### Premature cell death in the absence of vMIA

**Figure 1:**
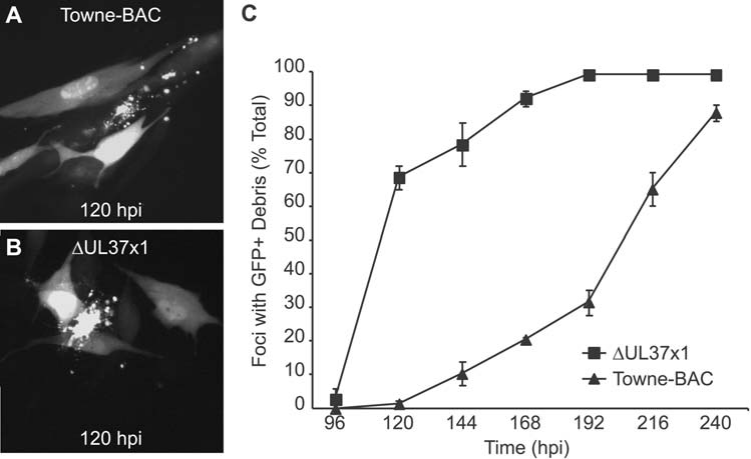
Pattern and timing of cmvPCD in Towne-BAC and ΔUL37x1-infected cells. Images of infected cell foci (multiplicity of infection [MOI] of 0.01) showing cmvPCD in Towne-BAC (A) and ΔUL37x1 (B) infected cells at 120 h postinfection (hpi). GFP expression by the viruses was used to identify foci of infected cells. Original magnification ×400. (C). The mean percentages (±standard deviation) of GFP+ foci with fragmented cells based on counting 400 infected foci each day. Infection was with a MOI of 0.0001. The mean±sd is depicted in all figures, except where indicated.

We evaluated cmvPCD during wild type (wt) virus (Towne-BAC, a GFP-expressing virus [44]) infection by scoring morphological changes in cells during replication (Fig. 1, supplemental Fig. S1). Termination of infection was associated with the accumulation of GFP-positive cell debris that remained associated with the monolayer (Fig. 1A). Cell fragmentation and death was first observed at 120 h postinfection in a small percentage of GFP+ foci (Fig. 1A, Fig. S1). GFP+ dead cell debris was observed only in foci (Fig. S1). Almost all (>90%) foci showed evidence of fragmentation by 240 h postinfection (Fig. 1C). Thus, cmvPCD occurred very late in infection, after maturation and release of progeny virus had peaked. GFP+ debris was observed much earlier during infection with vMIA null mutant virus, ΔUL37x1 (Fig. 1C), although the fragmentation process appeared similar to wt virus (Fig. 1B, Fig. S1). As previously reported [5], a majority (70%) of mutant virus foci contained debris by 120 h due to the single GFP+ cells that started to fragment between 72 and 96 h postinfection prior to the formation of foci (Fig. S1). There was a gradual increase in foci containing fragmented cells between 120 and 192 h postinfection such that, by 192 h, >90% of foci contained fragmented cells (Fig. 1C). This was consistent with our previous report showing both viruses spread with equivalent efficiency but that ΔUL37x1 induced premature caspase-independent death [5].

### ΔUL37x1-initiated death is a late phase event

**Figure 2:**
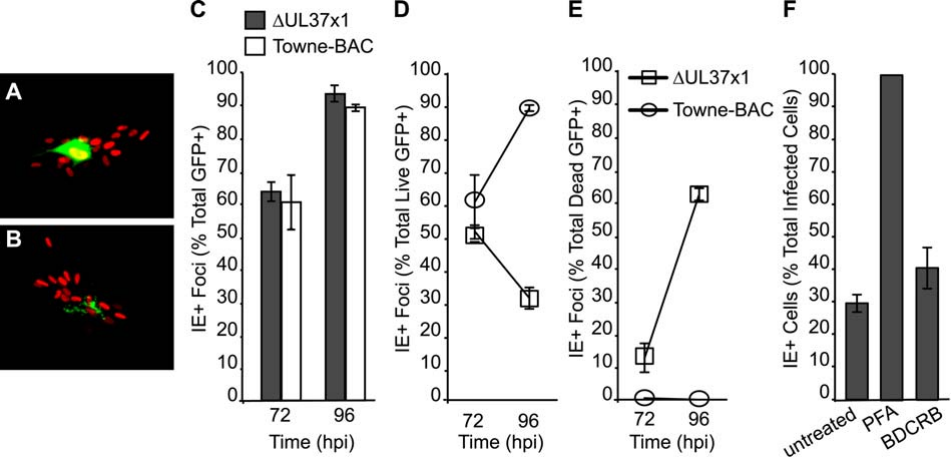
cmvPCD follows maturation and release in Towne-BAC and ΔUL37x1-infected cells. Representative fluorescent images of immediate early positive (IE+) nuclei in foci surrounding an intact, live (A) or fragmented, dead GFP+ cell (B). (GFP = green, indirect fluorescence of IE antigen = red; original magnification ×200). (C–E) Percentage of total GFP+ (C), live GFP+ (D), or dead (debris) (E) GFP+ cells forming IE1+ foci at 72 and 96 h postinfection with ΔUL37x1 or Towne-BAC (MOI of 0.001). A total of 300 GFP+ cells per virus were evaluated at each time for the experiment depicted. (F) Percentage of IE+ cells (live) at 96 h postinfection with ΔUL37x1 (MOI of 0.003), comparing untreated cultures to cultures treated with 300 µg/ml PFA or 20 µM BDCRB from 1 h postinfection. Data are from a representative experiment in which a total of >1000 infected cells/foci were evaluated for each condition.

Intact GFP+ ΔUL37x1-infected cells appeared to fragment before virus spread to form foci (Fig. 1C, Fig. S1) [5]. To determine whether intact ΔUL37x1-infected cells released progeny virus before fragmenting, we evaluated the formation of immediate early nuclear antigen positive (IE+) foci by immunofluorescence. Infected cells became IE+ earlier than they became GFP+ (Fig. 2). IE+ foci surrounding single GFP+ cells were detected at 72 h postinfection (Fig. 2A–E), when a majority (>50%) of GFP+ cells were still intact (Fig. 2A, 2D). Whether intact or fragmented, >99% of ΔUL37x1-infected cells produced foci by 120 h postinfection [5]. Thus, most virus spread occurs before cells fragmented (Fig. 2A, 2D), suggesting that, like wt, mutant virus is released before infected cells die (Fig. 1, Fig. S1).

To confirm that the death of mutant virus-infected cells was dependent on late phase events, the DNA synthesis inhibitor phosphonoformate (PFA) or the DNA encapsidation inhibitor 2-bromo-5,6-dichlorobenzimidazole (BDCRB) was added at the time of CMV infection and cell fate was scored by staining for IE+ cells at 96 h postinfection (Fig. 2B, 2F). PFA inhibits late gene expression, including GFP, while BDCRB blocks virion maturation but allows late gene expression to proceed [45],[46]. Untreated control or BCDRB-treated infected cultures appeared similar, whereas PFA-treated cultures contained single IE+ cells that remained GFP-. Only about 30% of GFP+ cells or debris in untreated ΔUL37x1-infected cells were IE+ at 96 h postinfection (Fig. 2F). The remaining 70% were no longer IE+, suggesting that IE expression was lost as cells fragmented. BDCRB-treated cells exhibited a pattern similar to untreated cells, suggesting that fragmentation was triggered by events prior to DNA encapsidation. All infected cells in PFA-treated cultures remained IE+ (Fig. 2F) and did not fragment, suggesting that initiation of cell death was dependent on events that followed viral DNA synthesis. Thus, initiation of death was dependent on cellular changes associated with viral DNA replication and/or late phase gene expression.

### cmvPCD proceeds rapidly through fragmentation to death

**Figure 3:**
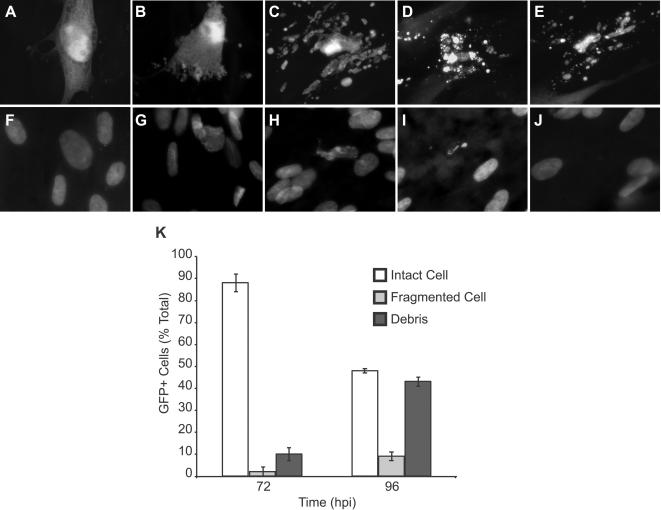
cmvPCD proceeds through cell fragmentation and nuclear collapse in the absence of chromatin condensation. Representative images showing stages of infected cell fragmentation and death in ΔUL37x1 infected cultures (collected at 72 h postinfection). Fluorescent micrographs of GFP+ virus-infected cells (A–E) and Hoechst-stained nuclei (F–J) associated with fragmentation. (A, F) Cytomegalic infected cell without signs of fragmentation, (B, G) initial stages of fragmentation with intact nucleus, (C, H) fragmented cell body with collapsed nuclear body, (D, I) fragmented cell body with residual nuclear body, and (E, J) fragmented cell debris without residual nuclear material. Original magnification ×1000. (K) Counts of GFP+ intact infected cells (Intact Cell), Hoechst positive fragmented cell bodies (Fragmented Cell), and Hoechst negative fragmented cell debris (Debris) at 72 and 96 h postinfection with ΔUL37x1 (MOI of 0.002). Data are from a representative experiment in which a total of 750 infected cells/foci were evaluated for each time.

We evaluated cell morphology [11] associated with death in ΔUL37x1 and wt virus-infected cells (Fig. 3). Late in CMV infection, inclusions form within enlarged cells coincident with replication and maturation processes that take place in the nucleus as well as in the cytoplasm. At 72 h postinfection, ΔUL37x1 and wt virus-infected cells exhibited similar nuclear and cytoplasmic inclusions [1],[47],[48] as well as enlarged cell CPE (Fig. 3A, 3F and Fig. S2) [5]. Stain for total nuclear DNA revealed a diffuse pattern (Fig. 3F, Fig. S2) that became distorted (Fig. 3G) and progressed through shrinkage and collapse and loss of nuclei (Fig. 3H–J) as cells fragmented (Fig. 3C–E). A similar process accompanied fragmentation in mutant or wt infected cells. The fragmentation process produced cell debris (Fig. 3E) lacking signs of DNA (Fig. 3J). Loss of nuclei scored by DNA stain or IE antigen (Fig. 2) was similar. Cell debris remained GFP+ and unstained by ethidium homodimer (Fig. S3), suggesting a non-necrotic death. Fragmentation was not synchronous in infected cultures, such that only 10% of infected cells exhibited intermediate fragmentation patterns (e.g. Fig. 3B–D) at any time (Fig. 3K and Fig. S1). The same types of morphological changes that started at 72 h in ΔUL37x1-infected cultures started at 120 h postinfection in Towne-BAC-infected cells (Fig. S1 and S4). The fragmentation of GFP+ cells (Fig. 1), loss of IE+ cells (Fig. 2) and loss of DNA+ nuclei (Fig. 3) were all characteristic of cmvPCD in wt and premature death in mutant virus-infected cells.

### Serine proteases induce cmvPCD

**Figure 4:**
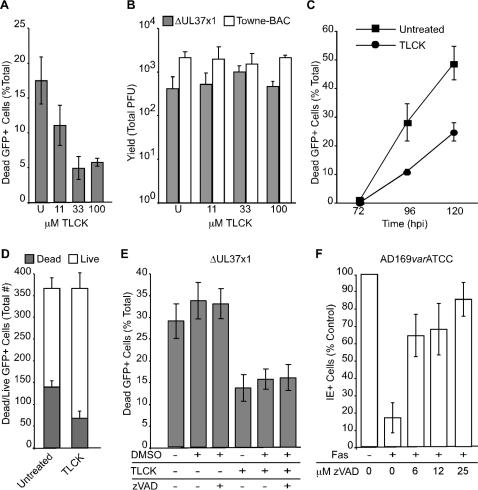
The serine protease inhibitor TLCK prevents ΔUL37x1-induced premature cmvPCD. (A) Percentage of dead GFP+ infected cells at 72 h postinfection in untreated cultures (U) or cultures treated with 11, 33, or 100 µM TLCK from 30 h postinfection with ΔUL37x1 (MOI of 0.001). A total of 300 cells/foci were evaluated at each condition. (B) Virus yield at 120 h postinfection from cell cultures left untreated (U) or treated with 11, 33, or 100 µM TLCK from 30 h postinfection with ΔUL37x1 or Towne-BAC (MOI of 0.001). (C) Percentage of fragmented GFP+ cells at 72, 96, or 120 h postinfection with ΔUL37x1 (MOI of 0.005) in control cultures or cultures treated with 33 µM TLCK from 30 h postinfection. A total of 1450 cells/foci were evaluated at each condition. (D) Absolute numbers of live or dead GFP+ cells at 96 h postinfection with ΔUL37x1 (MOI of 0.001) in untreated cultures or cultures treated with 33 µM TLCK from 30 h postinfection. (E) Percentage of dead GFP+ cells at 96 h postinfection with ΔUL37x1 (MOI of 0.005) in untreated cultures or cultures treated with 33 µM TLCK, 25 µM zVAD.fmk, or 0.05% DMSO, added from 30 h postinfection. A total of 300 cells/foci were evaluated at each condition. (F) Percentage of live cells at 48 h postinfection with AD169*var*ATCC (MOI of 3) following treatment with anti-Fas antibody to induce apoptosis in control cultures or cultures treated with 6, 12, or 25 µM zVAD.fmk, added at 24 h postinfection.

Previous work showing that caspases, cathepsins, or calpains were not involved in ΔUL37x1-initiated premature death [5], lead us to evaluate the contribution of cellular serine proteases to this process. We started by assessing the impact of a broad-spectrum inhibitor, TLCK [49]–[62], because this inhibitor does not affect the viral maturational serine protease at concentrations that are sufficient to block cellular serine proteases [63]. Addition of TLCK (11, 33, or 100 µM) to infected cultures at 30 h lead to a concentration-dependent reduction in cell fragmentation at 72 h postinfection (Fig. 4A). These concentrations of inhibitor did not reduce virus yields (Fig. 4B). Thus, TLCK inhibited premature cell death without any impact on virus replication. When TLCK was added at 30 h and fragmented cells were counted at 72, 96, and 120 h postinfection (Fig. 4C), cell death was reduced approximately twofold, suggesting that serine proteases play a central role in the timing of fragmentation. Despite experiment-to-experiment variability in the levels and rate of fragmentation death observed between 72 and 120 h postinfection, TLCK consistently inhibited this process (Fig. 4A and C) and increased the proportion of live, intact cells while absolute numbers of GFP+ cells or debris remained the same (Fig. 4D). This result implicated serine proteases early in the premature death induced by mutant virus.

Previously, we reported that the pan-caspase inhibitor zVAD.fmk had no effect on ΔUL37x1-induced premature death [5]. To determine whether caspases influenced death levels when serine proteases were inhibited, we applied zVAD.fmk alone as well as in combination with TLCK. The caspase inhibitor did not influence the serine protease-dependent process (Fig. 4E). In contrast, zVAD.fmk showed the expected [5],[6],[64] inhibition of apoptosis induced in CMV strain AD169*var*ATCC infected cells (Fig. 4F). Thus, these data imply that cmvPCD is controlled by serine proteases that work independent of caspases.

### The serine protease HtrA2/Omi initiates cmvPCD

**Figure 5:**
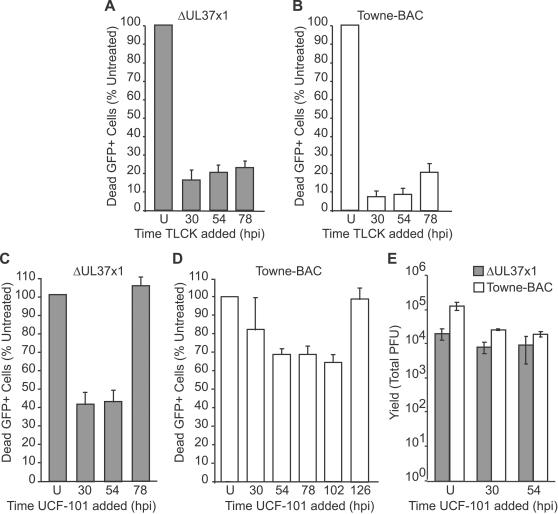
Inhibitors of serine protease HtrA2/Omi suppress cmvPCD. (A) Percentage of dead GFP+ cells at 96 h postinfection with ΔUL37x1 (MOI of 0.01) in cultures treated with 100 µM TLCK from 30, 54, or 78 h postinfection relative to untreated cultures (U). 3000 cells/foci were evaluated at each condition. (B) Percentage dead GFP+ cells at 144 h postinfection with Towne-BAC (MOI of 0.03). 9000 cells/foci were evaluated at each condition. (C) Percentage of dead GFP+ cells at 96 h postinfection with ΔUL37x1 in cultures treated with 10 µM UCF-101 from 30, 54, or 78 h postinfection relative to untreated cultures (U). 3000 cells/foci were evaluated at each condition. (D) Percentage of dead GFP+ cells at 144 h postinfection with Towne-BAC (MOI of 0.03). A total of 9000 cells/foci were evaluated at each condition. Data in A–D is depicted relative to untreated controls, which were assigned a value of 100%. (E) Yields of virus at 168 h postinfection (MOI of 0.01) with ΔUL37x1 or Towne-BAC in untreated cultures or cultures treated with UCF-101 from 30 or 54 h postinfection.

To determine the timing of serine protease activity in controlling premature death, TLCK was added to ΔUL37x1-infected cells at 30, 54, or 78 h. Addition of TLCK at each of these times was found to dramatically reduce the level of death at 96 h postinfection (Fig. 5A). These results suggest serine proteases act within 24 h of fragmentation (Fig. 4D) and demonstrated the importance of these proteases late in infection. Taken together with data on timing of the death stimulus (Figs. 2 and 3, and [5]), serine proteases active late in CMV infection may either trigger or play an intermediary role in the cell death pathway. To determine the timing of serine protease activity in wt virus-induced cmvPCD, TLCK was added at 30, 54, and 78 h (Fig. 5B). Addition of TLCK at each of these times effectively reduced cell fragmentation at 144 h postinfection, suggesting that proteases active after the 78 h time period played a critical role during wt virus infection as well (Fig. 5B). These data demonstrate a common serine protease cell death pathway terminates mutant or wt virus infection, and demonstrate that the premature death in mutant virus infected cells follows a similar pathway as cmvPCD. Differences in timing show the importance of vMIA control in the timing of cmvPCD.

One mitochondrial serine protease, HtrA2/Omi, has been implicated in cell death pathways and exhibits sensitivity to TLCK [40], [65]–[67]. The specific HtrA2/Omi inhibitor UCF-101 [68] was added to ΔUL37x1- or Towne-BAC-infected cultures (Fig. 5C–E) at a concentration (10 µM) anticipated to minimize a previously recognized impact on other cellular targets [69]. UCF-101 reduced death when added to ΔUL37x1 or Towne-BAC-infected cultures at 30 or 54 h, implicating HtrA2/Omi as a mediator of cmvPCD (Fig. 5C–D). Although UCF-101 added at 54 h reduced death of ΔUL37x1-infected cells at 96 h postinfection, addition at 78 h was ineffective (Fig. 5C). Towne-BAC-associated death at 144 h was reduced by UCF-101 added as late as 102 h, but not when added at 126 h (Fig. 5D). UCF-101 treatment did not reduce viral yields (Fig. 5E). Most importantly, these data suggest that events over the 24 to 48 h preceding fragmentation of cells are influenced by HtrA2/Omi, regardless of whether considering the premature cmvPCD in mutant virus infected cells or cmvPCD in wt infection. The differences between UCF-101 and TLCK addition at 78 h (Fig. 5A) may be due to the effectiveness of these inhibitors on HtrA2/Omi or to additional serine proteases that contribute to cmvPCD. Overall, these data demonstrate that UCF-101 specifically reduces infected cell death and implicate the serine protease HtrA2/Omi in the pathway. Further, these data implicate HtrA2/Omi as a target of vMIA modulation.

### HtrA2/Omi levels increase late in infection

**Figure 6:**
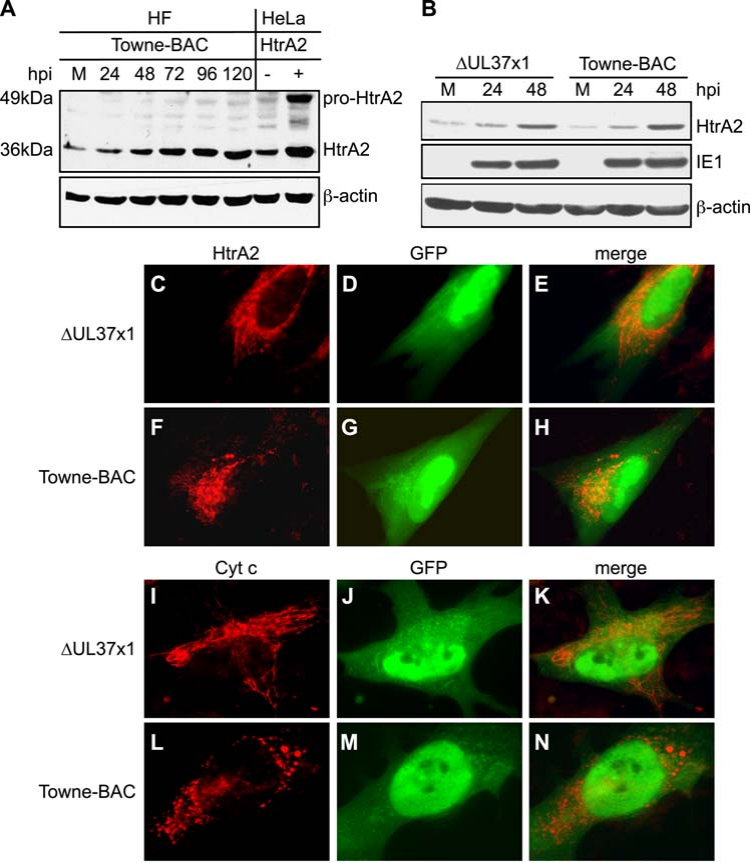
HtrA2/Omi expression increases following CMV infection. (A–B) Immunoblot analyses of HtrA2/Omi in lysates of mock-infected HF (M) or HF infected (MOI of 3) with Towne-BAC or ΔUL37x1 for 24, 48, 72, 96, or 120 h and control cell lysates from HeLa cells transfected with HtrA2/Omi expression plasmid (+) or control plasmid (−). The expected migration of the 49 kDa immature, pro-HtrA2/Omi and 36 kDA mature, HtrA2/Omi was calculated based on migration of molecular weight markers (not shown). Immunoblot detection of HtrA2, β-actin and IE1 are shown. (C–N) Immunofluorescent images of HtrA2/Omi (C, F) and cytochrome *c* (I, L) (red), and GFP fluorescence (green) (D, G, J, M) and merged images (E, H, K, N) at 72 h (C–H) or 96 h (I–N) postinfection (MOI of 0.001) with ΔUL37x1 (C–E, I–K) or Towne-BAC (F–H, L–N). Original magnification ×1000.

To determine the impact of mutant or wt virus infection on HtrA2/Omi expression levels and subcellular localization as well as to investigate any impact of vMIA on HtrA2 expression, immunoblot analysis was carried out on Towne-BAC infected cells (Fig. 6). Levels of mature 36 kDa HtrA2/Omi levels were similar to uninfected cells at 24 h, but increased by 48 h and continued to accumulate over the course of infection (Fig. 6A–B). Comparisons of mutant and wt infected cells showed similar accumulation of HtrA2 by 48 h (Fig. 6B, Fig. S5). Premature cmvPCD initiated in mutant virus-infected cells prevented comparisons by immunoblot later in infection; however, immunofluorescence analyses at 96 h postinfection confirmed the dramatically increased HtrA2/Omi levels in mutant or wt virus-infected cells (Fig. 6C–H and Fig. S6). HtrA2/Omi colocalized with the mitochondrial membrane potential marker MitoTracker Red (Fig. 6C, 6F and Fig. S6) at late times of infection with either virus. These data indicate that HtrA2/Omi levels increase within mitochondria before the initiation of cmvPCD. vMIA does not alter expression pattern or mitochondrial localization of this protease but nevertheless prevents death.

Mitochondria in wt CMV infected cells followed the expected [22] reticular to punctate transition associated with disruption of mitochondrial networks (Fig. 6F, 6L, and Fig. S6) and mutant virus-infected cells retained a reticular pattern (Fig. 6C, 6I and Fig. S6) when stained for HtrA2/Omi, cytochrome *c*, mitochondrial HSP (mtHSP70), or MitoTracker Red. MitoTracker Red staining indicated that mitochondria retained a similar membrane potential despite this difference in morphology due to vMIA (Fig. S6). When the kinetics of the reticular to punctate transition was evaluated in Towne-BAC-infected cells, almost all (≥90%) of cells contained reticular mitochondria at 48 h, but transitioned to punctate by 96 h. In contrast, ΔUL37x1-infected cells retained a reticular morphology (≥80%) throughout infection. These data suggest that a vMIA-dependent process disrupts reticular mitochondria beginning at 48 h postinfection and this change in mitochondrial organization may contribute to cell survival. Despite this striking difference in mitochondria, the organelles of the secretory apparatus that form the viral assembly compartment at late times of infection [48],[70] were similar in either virus infection (Fig. S2, Fig. S6). Thus, disruption of mitochondrial networks by wt virus may contribute to control of HtrA2/Omi-dependent death and the failure of mutant virus to induce these changes may lead to premature HtrA2/Omi-dependent death.

We sought to determine whether mitochondria released cytochrome *c* prior to premature cmvPCD. Cells that had not yet started to fragment all showed reticular cytochrome *c* staining (Fig. 6 and Fig. S6) whereas diffuse staining was detected only as cells became highly fragmented (Fig. S7). These data suggest that release of cytochrome *c* follows the fragmentation that characterizes cmvPCD.

### HtrA2/Omi overexpression early after infection blocks CMV replication

**Figure 7:**
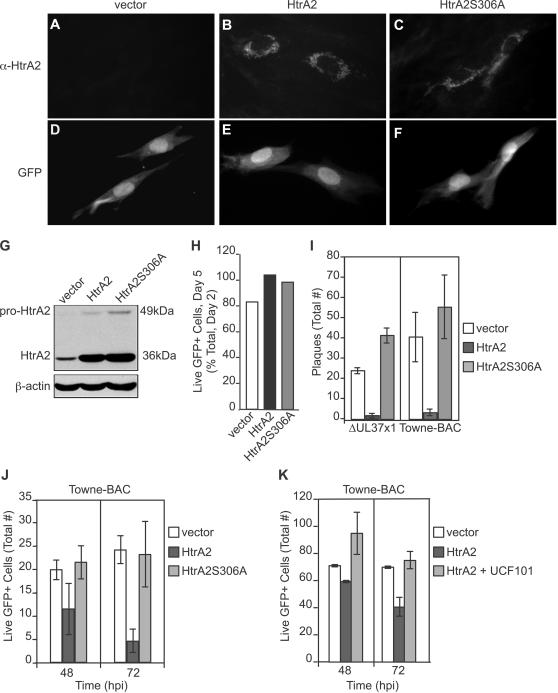
Transient overexpression of HtrA2/Omi induces early death in CMV-infected but not uninfected cells. (A–F) Immunofluorescent images of HtrA2/Omi (A–C) and GFP fluorescence (D–F) following cotransfection of HF with GFP expression plasmid together with empty vector, HtrA2/Omi, or HtrA2S306A expression plasmids. Original magnification ×400. (G) Immunoblot depicting 49 kDa, immature and 36 kDA, mature forms of HtrA2/Omi and HtrA2S306A as well as β-actin control in transfected HeLa cell lysates. (H) Percentage of live HFs at 120 h post cotransfection of GFP expression plasmid with vector, HtrA2/Omi, or HtrA2S306A expression plasmids. (I) Number of viral plaques 10 days following cotransfection of ΔUL37x1 or Towne-BAC DNA (500 ng) with 800 ng vector, HtrA2/Omi, or HtrA2S306A expression plasmids. (J) Number of live (intact) cells in cultures at 48 or 72 h following transfection with Towne-BAC DNA (300 ng) alone or with 333 ng of HtrA2 or HtrA2S306A expression plasmids. (K) Number of live (intact) cells (±range) at 48 or 72 h post transfection with Towne-BAC DNA (500 ng) and 300 ng of vector or HtrA2/Omi expression plasmid with or without addition of 10 µM UCF-101 from 6 h.

To directly address the impact of HtrA2/Omi overexpression on the cell fate, full-length HtrA2/Omi as well as a catalytic site mutant (HtrA2S306A) [34] were transiently expressed in uninfected and virus-infected cells. Initially, expression levels and impact on uninfected cell viability were assessed (Fig. 7A–H, Fig. S8). HtrA2/Omi (or mutant HtrA2/Omi) overexpression did not induce death in uninfected HFs (Fig. 7H) or HeLa cells (Fig. S8), consistent with published characterization of full-length protease [37]. Immunofluorescence patterns revealed the expected mitochondrial localization at 48 h post transfection (Fig. 7A–F), and immunoblot analyses using HeLa cells indicated equivalent expression levels of the wt and mutant protease (Fig. 7G). To determine the impact of HtrA2/Omi overexpression on infected cells, Towne-BAC or ΔUL37x1 were cotransfected with HtrA2/Omi or HtrA2S306A expression plasmids (Fig. 7I) and assessed for spread to form foci [71]. By 10 days posttransfection, wt and mutant BACmids had produced comparable numbers of plaques, as expected [5]. Cotransfection of HtrA2/Omi expression plasmid reduced the plaquing efficiency >10-fold compared to vector control or HtrA2S306A mutant (Fig. 7I). These data show that overexpression of catalytically active HtrA2/Omi prevents plaque formation independent of vMIA expression.

To determine whether the reduction in plaguing efficiency following overexpression of HtrA2/Omi was due to cell death induction, the fate of individual cells was monitored (Fig. S9). When Towne-BAC was cotransfected with HtrA2/Omi or HtrA2S306A, individual GFP+ cells were observed at 48 h, although even at this time the levels could be lower in cells receiving the protease active form (Fig. 7J–K). HtrA2/Omi/GFP+ cells began to fragment by 72 h posttransfection (Fig. 7J) and were lost from cultures by 168 h (Fig. S9). HtrA2/Omi overexpression-induced death required the active protease, based on the failure of HtrA2S306A to induce death (Fig. 7J) as well as on the ability of the inhibitor UCF-101 to block HtrA2/Omi overexpression-induced death (Fig. 7K). The numbers of GFP+ cells (Fig. 7J) or plaques (Fig. 7I) that formed following cotransfection of Towne-BAC with HtrA2S306A could not be distinguished from transfection of Towne-BAC with vector. These data show that overexpression of catalytically active HtrA2/Omi induces infected cell death that is independent of vMIA expression. The sensitivity of virus-infected cells and lack of impact on uninfected HFs (Fig. 7H) supports the specific role of HtrA2/Omi in a novel cell death pathway in CMV-infected cells.

**Figure 8:**
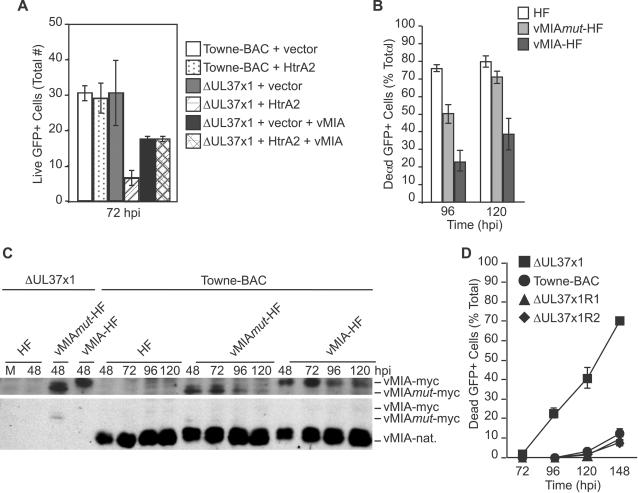
vMIA suppression of HtrA2/Omi-dependent cmvPCD. (A) Average number of live cells (±range) in cultures at 72 h post transfection of Towne-BAC DNA (500 ng) with 12 ng of HtrA2/Omi expression plasmid or control vector or post transfection of ΔUL37x1 DNA (500 ng) with 12 ng of HtrA2/Omi expression plasmid in the absence or presence of 15 ng vMIA-encoding plasmid or 12 ng of control vector in the absence or presence of 15 ng vMIA-encoding plasmid. (B) Percentage of dead cells at 96 and 120 h postinfection (MOI of 0.0001) with ΔUL37x1 in HF, vMIA*mut*-HF, or vMIA-HF cultures. 400 cells/foci were evaluated for each condition. (C) Immunoblot detection of native vMIA (vMIA-nat.), vMIA-myc, and vMIA*mut*-myc in cell lysates collected at various h postinfection (MOI of 3) with ΔUL37x1 or Towne-BAC. Top, α-myc antibody; bottom, α-vMIA antibody. (D) Percentage of dead cells at 72, 96, 120, and 148 h postinfection (MOI of 0.0001) with ΔUL37x1, Towne-BAC, ΔUL37x1R1, or ΔUL37x1R2. 100 cells/foci were evaluated for each condition.

A role of vMIA in HtrA2-induced death was investigated using the cotransfection assay carried out using lower doses of expression plasmids as well as using vMIA-expressing cells. Cotransfection of HtrA2/Omi expression plasmid at a 25 or 30-fold lower level revealed a differential impact on these viruses (Fig. 8A), where Towne-BAC exhibited a greater resistance to HtrA2/Omi-induced death. These conditions were also employed to demonstrate that vMIA overexpression overcame HtrA2/Omi-induced death (Fig. 8A). To address the role of vMIA further, HFs as well as HFs stably transduced with retroviruses expressing Myc-tagged vMIA or mutant protein [18] vMIA*mut* (Fig. 8B–C) were infected. As expected [5], vMIA-HFs suppressed the premature cmvPCD when assessed at 96 and 120 h postinfection, whereas vMIA*mut*-HF or nontransduced control HFs did not (Fig. 8B). These data suggest the intact antiapoptotic domain of vMIA is required to control premature cmvPCD. The experimental plating efficiency of ΔUL37x1 virus was the same on either cell line (Fig. S10 and [5]). These results were consistent with a role for vMIA in controlling kinetics of cmvPCD and suggest that similar functional domains of vMIA are required in suppression of apoptosis or HtrA2-dependent cmvPCD. Immunoblot analyses were used to compare transduced vMIA (or vMIA*mut*) levels relative to native viral expression (Fig. 8C). The lower levels of transduced gene expression likely contribute to the death suppression observed (Fig. 8B). Rescue viruses derived from ΔUL37x1 confirmed that an intact UL37x1 locus is sufficient to completely control premature death, mitochondrial organization, and viral yield (Fig. 8D, Fig. S10). Overall, these data confirm the critical role of vMIA as a determinant of cmvPCD when induced by overexpression of HtrA2/Omi transfection or during the late phase of infection. Thus, ΔUL37x1 infection sensitizes to the prodeath impact of HtrA2/Omi, and vMIA controls HtrA2/Omi prodeath pathways during wt CMV infection.

### vMIA antiapoptotic function remains intact and independent of HtrA2/Omi during infection

**Figure 9:**
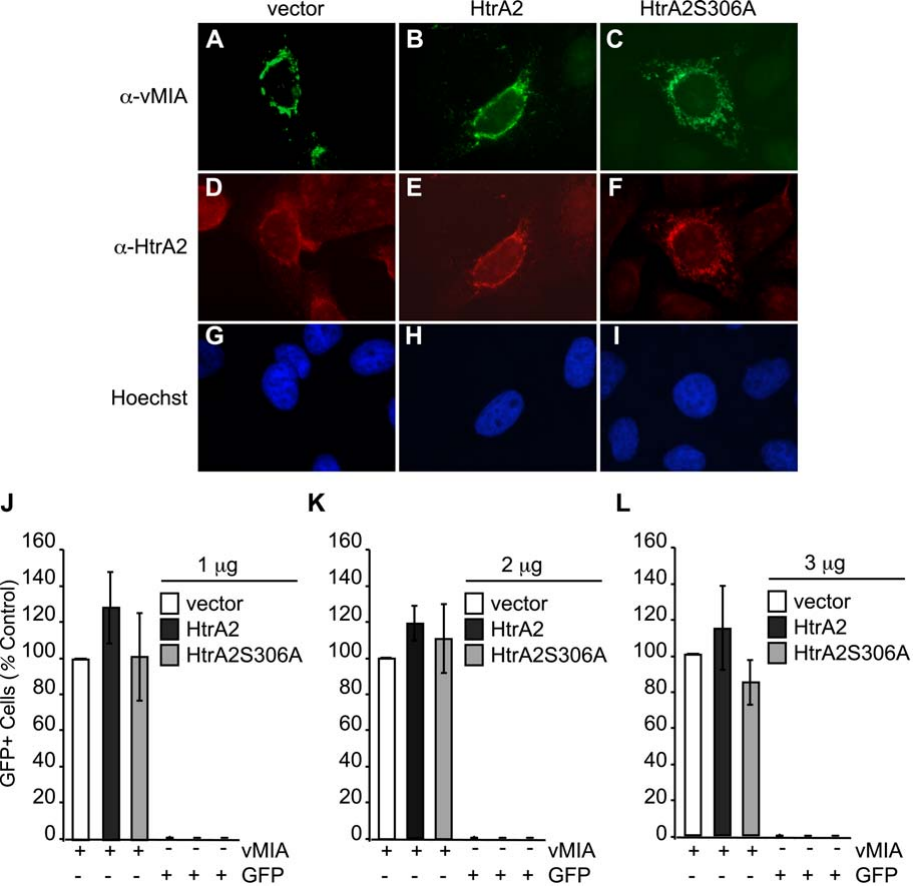
HtrA2/Omi overexpression does not impede vMIA antiapoptotic activity. (A–I) Immunofluorescent images of vMIA (A–C) or HtrA2/Omi (D–F) as well as Hoechst staining (G–I) in HeLa cells following transfection with vMIA together with vector, HtrA2/Omi, or HtrA2S306A expression plasmids. Original magnification ×400. (J–L) GFP+ cell number as a percentage of control wells (vector) of HeLa cells cotransfected with vMIA (1 µg) or GFP expression plasmids and 1 µg (J), 2 µg (K), or 3 µg (L) quantities of vector, HtrA2/Omi, or HtrA2S306A DNA. GFP+ cell numbers in vMIA plus vector wells were assigned values of 100%.

In order to determine whether the antiapoptotic activity of vMIA is preserved in cells where HtrA2/Omi is overexpressed, we performed experiments with HtrA2/Omi expression constructs in HeLa cells exposed to Fas-mediated apoptosis (Fig. 9) [2],[19]. Immunofluorescence analyses showed expected levels and localization of HtrA2/Omi and vMIA in transfected cells (Fig. 9A–I). These analyses indicated that vMIA and HtrA2/Omi (or HtrA2S306A) colocalize with mitochondria under all conditions. Introduction of HtrA2/Omi or mutant expression constructs did not influence the antiapoptotic activity of vMIA (Fig. 9J–L), consistent with previous work showing vMIA-dependent antiapoptotic function is active at late times of infection [5],[6]. Together, these data suggest that HtrA2/Omi does not interfere with vMIA-mediated control of apoptosis.

### Increased serine protease accumulation in cells undergoing premature cmvPCD

**Figure 10:**
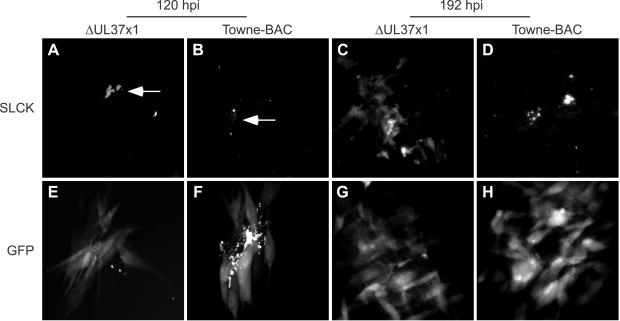
SLCK localization within infected cells. Images of fluorescent serine protease substrate SLCK localization (A–D) and GFP fluorescence (E–H) in ΔUL37x1 (A, C, E, G) or Towne-BAC (B, D, F, H) foci undergoing fragmentation at 120 h (A–B, E–F) and 192 h (C–D, G–H) postinfection (MOI of 0.001). Arrows indicate SLCK-bound debris. Original magnification ×400 (A–H).

To directly visualize levels of serine proteases in infected cells, the fluorescent reagent sulforhodamine 101-leucine chloromethyl ketone (SLCK) was used to reveal the distribution and activity of serine proteases [72] in ΔUL37x1 or Towne-BAC infected live cell cultures (Fig. 10). By day 5, foci with brightly stained GFP+ debris was observed in cultures infected with either virus (Fig. 10E–F), although fragmentation was rare in wt virus-infected cultures at this time. The SLCK staining pattern was distinct in ΔUL37x1-infected cells and included bright SLCK+ debris (Fig. 10A) that was distinguishable from Towne-BAC infected cells by differences in the amount of staining as well as the size and distribution of debris (Fig. 10A–B). By 8 days after infection, most ΔUL37x1-infected cells in each plaque were brightly fluorescent (Fig. 10C) whereas cells infected with wt virus (Fig. 10D) showed only SLCK+ debris. SLCK staining patterns did not appear to be mitochondrial at any time in either virus infection. These patterns were distinct from HtrA2/Omi (Fig. 6C), suggesting that SLCK labeling detected serine proteases in addition to HtrA2/Omi. Addition of 0.1 or 1 mM TLCK reduced but did not eliminate SLCK binding to mutant virus-infected cells, consistent with the induction of serine proteases (Fig. S11). Overall, >50% of GFP+ cells in ΔUL37x1 plaques also labeled with SLCK. SLCK staining was reduced to ≤30% by addition of 100 µM TLCK. Thus, SLCK revealed a higher level of protease activation in CMV infected cells that were susceptible to premature cmvPCD. This data suggests that vMIA may control a broader serine protease-dependent death pathway by counteracting mitochondrial HtrA2/Omi during viral infection.

## Discussion

CMV replicates in the nucleus, matures in the cytoplasm and is released into the surrounding medium or adjacent cells over the course of a 7 to 10 day replication cycle [1]. Host cells are dramatically reprogrammed for production of progeny virus until death occurs via a process that begins late in CMV infection, associated with late gene expression that drives CPE and cell cycle dysregulation [73]–[76]. In an effort to define viral and cellular contributions to morphological and biochemical events that terminate CMV infection, we have discovered the key role of mitochondrial HtrA2/Omi and a novel cell death pathway. This cellular serine protease appears to be responsible for induction of cmvPCD following a pathway that is held in balance by the viral cell death suppressor, vMIA. vMIA resides in the mitochondrion where it is a potent suppressor of cytochrome *c* release, thereby preventing activation of executioner caspases during apoptosis [2]–[4]. In addition to suppression of apoptosis, vMIA carries out a distinct and nonoverlapping role suppressing death induced by HtrA2/Omi during the late phase of viral infection. This cmvPCD pathway is triggered only in the context of infection. Late phase infected cell events promote cell fragmentation together with collapse, shrinkage, and loss of nuclei in a pathway that is dependent on HtrA2/Omi protease activity and associated with the activation of cytoplasmic serine proteases that may act as executioners. HtrA2/Omi levels rise before induction of death, consistent with a central role of this protease in initiation of cmvPCD. Suppression of this death pathway, like suppression of apoptosis, is associated with global disruption of mitochondrial networks by vMIA. Unlike apoptosis, however, cmvPCD apparently does not require cytochrome *c* release from mitochondria to trigger downstream events. Further, HtrA2/Omi remains mitochondrial late during infection suggesting death may be initiated by the activity of the intramitochondrial protease, which raises an interesting question as to how transduction of the death signal occurs. Our data reveal a pathway that is triggered by high intramitochondrial HtrA2/Omi protease and controlled by vMIA.

Although vMIA-mutant virus undergoes premature cmvPCD, the fragmentation process is similar to cmvPCD in wt virus-infected cells. The difference appears to be in timing of cell death. vMIA delays death for several days beyond the initiating trigger which is coincident with the late phase of replication. Although induction of HtrA2/Omi is independent of vMIA, the impact of induction appears to be the target of vMIA function at the mitochondria where both reside. Suppression of cmvPCD benefits the virus by extending the period of virus production by infected cells [5], although cultured fibroblasts show only slight reduction in yield and cell-to-cell transmission in the absence of vMIA. A prolonged period of virus production increases the amount of virus released cell-free and potentially benefits transmission in natural settings. A delay in fragmentation would also delay phagocytosis and clearance of virus-infected cells [77]. HtrA2/Omi-dependent death may be viewed as an intrinsic host antiviral process analogous to apoptosis. vMIA control of HtrA2/Omi-mediated death is analogous to control of apoptosis, as both appear to be independent cell-intrinsic mechanisms of pathogen control. Importantly, vMIA appears to provide concurrent protection from both pathways.

vMIA disruption of reticular networks and organization of mitochondria [22] is independent of HtrA2/Omi accumulation within mitochondria, but does correlate with cell death suppression activity. Thus, ΔUL37x1-mutant virus-infection preserves mitochondrial networks throughout infection, during HtrA2/Omi accumulation and initiation of premature cmvPCD. In contrast, wt virus infected cells support the same accumulation of HtrA2/Omi and a vMIA-driven disruption of mitochondrial networks but survive. The correlation between this vMIA-dependent disruption and cell death protection suggests that punctate mitochondria may be more resistant to the stress induced by late phase events. Reticular mitochondria are known to rapidly disseminate Ca^++^ or ATP signals; whereas, punctate mitochondria have slower responses to changes in intracellular mediators [78]. Additional experiments will be needed to understand the mechanism underlying resistance of punctate mitochondria to death, whether mediated via caspases or HtrA2/Omi.

Emerging evidence suggests vMIA, viral strain differences, and cellular factors contribute to the control of mitochondria and death. Thus, AD-BAC kills cells earlier [6] and disrupts mitochondrial networks by 24 h postinfection [22] whereas Towne-BAC disruption occurs later, by 48 h postinfection and cells die later. vMIA associates with the outer mitochondrial membrane within 24 h [79],[80]. AD-BAC (or its parent AD169*var*ATCC) depends upon vMIA to suppress caspase-dependent apoptosis that develops by 48 to 72 h postinfection. Towne-BAC (or its parent Towne*var*ATCC) depends on vMIA to suppress caspase-independent, HtrA2-dependent cell death that develops by 72 to 96 h postinfection. It remains to be seen whether vMIA suppresses both pathways in cells infected with strains like AD-BAC. Accumulation of HtrA2/Omi occurs in other viral strains (McCormick, unpublished), underscoring the overall importance of the process described here. There are many potential factors contributing to qualitative or quantitative differences in the way characterized viral strains initiate and control death, with apoptosis apparently predominating in some settings and HtrA2-mediated death predominating in others. We focused here on dissecting the novel death pathway in Towne-BAC-infected cells, to characterize a novel HtrA2/Omi pathway that is independent of apoptosis.

cmvPCD may be influenced by or even associated with a number of additional modulatory effects of this virus that impact late times of infection, including dysregulation of the cell cycle [73]–[76], disruption of p53 activation [81], DNA damage response [82],[83] and unfolded protein response [84] that all remain incompletely understood. vMIA may reduce ATP levels during infection as it does in established cell lines. Although suggested to control late CPE in AD-BAC derivatives [85] vMIA has no impact on development of CPE in Towne-BAC derivatives. Any vMIA-specific reduction in ATP levels is likely highly coordinated with other viral processes contributing to late CPE. CMVs encode multiple factors that target mitochondria [86],[87], regulate expression of mitochondrial proteins [75] and even stimulate mitochondrial DNA synthesis [88] suggesting viral control of mitochondria functions is complex. The vMIA-specific impact on ATP levels as related to HtrA2/Omi remains unknown but may certainly be a feature of control. As an event that occurs very late in replication, cmvPCD is crucial to sustaining viral infection in individual cells. Our observations that either mutation of vMIA or premature overexpression of HtrA2/Omi levels dramatically alters the timing of death indicate that these two may balance each another in controlling cmvPCD.

Previously, pharmacological inhibitor and overexpression studies have implicated HtrA2/Omi as a regulator of death [35],[37],[40],[89],[90]. Genetic studies have suggested this protease functions primarily to ensure normal mitochondrial homeostasis [39],[91],[92], perhaps controlling protein quality and cellular stress responses [34] similar to the related bacterial protease HtrA [41]–[43]. The role of HtrA2/Omi in caspase-independent cell death has not previously been studied in detail, although the truncated, active form of HtrA2 drives death when released from mitochondria or expressed directly in the cytoplasm [35],[37],[40],[90],[93]. We have shown that the active form drives death specifically and only in CMV-infected cells, which we correlate with the fact that the enzyme remains mitochondrial throughout CMV infection. CMV infection is a unique setting that has unveiled a direct role for HtrA2/Omi in a caspase-independent cell death pathway analogous to apoptosis.

vMIA controls the programmed death of infected cells after a week or more of replication, following a period of persistent virus production. CMV infects many cell types in addition to HFs, and given that the timing of replication varies with cell type, vMIA control of HtrA2/Omi-dependent death may be critical in other cell types or in natural infection of the human host. Given the many functions that CMV has evolved to manage the virus:host standoff, we speculate that viral control of cmvPCD represents a benefit to the virus, potentially allowing infected cells to avoid sending alarm signals. Other examples of viral proteins acting together to control the type of cell death that follows replication can be identified. Thus, the adenovirus death protein, ADP, functions in the presence of E1B-19k, the viral Bcl-2 protein, and both contribute to the type of death that terminates infection [94]–[96]. Caspase-dependent apoptosis is itself a cell-intrinsic pathogen clearance process, minimizing inflammation and pathology while alarming the immune system to initiate cellular responses [77]. CMV-encoded cell death suppressors provide a means of evading cell death directed by host cell intrinsic, innate, and adaptive responses [97]. The benefit of controlling the mechanism and timing of cell death includes persistence, as well as the interface of virus-infected cells with the host immune system. In the host, cmvPCD may provide for greater success in attaining a foothold without evoking clearance. The presence of vMIA-like functions in other cytomegaloviruses [5],[17] as well as the broad distribution of mitochondrial cell death suppressors in other viruses suggests this novel serine protease pathway may occur in other biological settings.

## Materials and Methods

### Accession numbers

The HtrA2 protease has MEROPS accession number S01.278 and the I.M.A.G.E. consortium clone obtained for these studies was identical in sequence to NCBI accession ID BC0000096. The vMIA [2] used to complement and repair ΔUL37x1 was obtained from AD169*var*ATCC genomic DNA; NCBI accession ID X17403. The sequence of Towne-BAC was deposited to NCBI [8]; accession ID AY315197.

### Cells and viruses

Human fibroblasts (HFs), vMIA-HFs, vMIA*mut*-HFs, and HeLas were cultured as previously described [5]. Viruses derived from the BACmid clones Towne-BAC and ΔUL37x1 [8] were maintained as DNA clones in *E. coli* or on complementing vMIA-HFs prior to experiments. AD169*var*ATCC was maintained as previously described [22].

### Generation of ΔUL37x1 rescue virus RC2707

The kanamycin selection cassette in ΔUL37x1 was replaced with UL37x1 sequence derived from AD169*var*ATCC to generate RC2707. Transfection of pON2707 [5] into HFs was followed by infection with ΔUL37x1 virus. Plaques that included cell death at a frequency similar to Towne-BAC virus [5] were isolated for further analysis. Sequencing of viral DNA from Towne-BAC and two independently derived isolates (ΔUL37x1R1, ΔUL37x1R2) confirmed replacement of the selection marker in ΔUL37x1 with UL37x1 nucleotide sequence identical to pON2707 and AD169*var*ATCC while the control, Towne-BAC, was identical to the expected sequence [8],[98]. Expression of vMIA was confirmed by immunoblot analysis.

### Plasmids

The HtrA2/Omi expression plasmid, pON601, was derived by restriction of the I.M.A.G.E. cDNA (HtraA2 clone #5344667, ATCC, Manassas, VA) with BsrGI, followed by removal of the single-stranded overhangs with Klenow DNA polymerase, and restriction with XhoI. The HtrA2/Omi encoding fragment was ligated to EcoRV and XhoI restricted pcDNA3.1+ (Invitrogen, San Diego, CA). The HtrA2S306A expression plasmid, pON602, was generated by PCR-site directed mutagenesis of the HtrA2 ORF to introduce the S306A mutation and a novel NaeI restriction enzyme site and utilized the mutagenic primer 5′-CTATTGATTTTGGAAACGCCGGCGGTCCCCTGGTTAAC-3′. Both clones were sequenced to confirm expected results. The vMIA and GFP expression clones and retroviral constructs used in these experiments were reported previously [2],[5],[18].

### Immunoblotting, immunofluorescence, and MitoTracker Red staining assays

Immunodetection employed mouse monoclonal antibodies to c-*myc* epitope (9E10; Santa Cruz Biotechnology, Santa Cruz, CA), HtrA2/Omi (MAB1458; R&D Systems, Inc, Minneapolis, MN), cytochrome *c* (Clone 7H8.2C12, BD Pharmingen, San Jose, CA), β-actin (AC-74, Sigma, St. Louis, MO), golgin-97 (CDF4; Molecular Probes, Eugene, OR), mitochondrial heat shock protein 70 (mtHSP70) (a gift from Susan Pierce, Northwestern University), viral nuclear antigens IE1_p72_ and IE2_p86_ (MAB 810, Chemicon, Temeculah, CA), ICP36 (CH16) and pp28 (CH19) (both from Virusys Corporation, Randallstown, MD) or rabbit polyclonal antiserum to native vMIA [2] and peroxidase-conjugated horse anti-mouse IgG or goat anti-rabbit IgG, Texas Red-conjugated horse anti-mouse IgG (all from Vector, Burlingame, Calif.), or AlexaFluor 350-conjugated goat anti-mouse IgG (Molecular Probes, Eugene, OR). Immunoblot analysis of total protein from infected cells and immunofluorescence assays followed previously described methods [22]. MitoTracker Red CMXRos (Molecular Probes, Eugene, OR) staining of mitochondria followed previously described methods [22].

### Detection of cmvPCD, impact of replication and protease inhibitors on death, viral yield, and BACmid transfections

To assess morphological changes in infected cells and nuclei, cells grown on coverslips and infected for varying periods of time were fixed with 3.7% formaldehyde, permeabilized with Triton X-100 (EMD Biosciences, Darmstadt, Germany), stained with Hoechst 33258 (AnaSpec, San Jose, CA), and processed for microscopic evaluation as previously described [22]. Some cultures were stained with ethidium homodimer 1 (Molecular Probes, Eugene, OR), as previously described [5] to assess virus-induced cell death.

Images from live cell cultures were obtained as previously described [5] or utilized Simple PCI software, a Retiga Exi digital camera, and a Leica DM IRB microscope. Imaging of cultures grown on coverslip employed an AxioCam MRc5 camera attached to a Zeiss Axio Imager.A1 and AxioVision Release 4.5 software.

Replication inhibitors included phosphonoformate (PFA Sigma, St. Louis, Mo) dissolved in water and 2-bromo-5,6-dichlorobenzimidazole (BDCRB from L. B. Townsend, University of Michigan) dissolved in dimethyl sulfoxide (DMSO) (Sigma, St. Louis, MO). Protease inhibitors included TLCK, N-alpha-p-tosyl-L-lysine chloromethyl ketone, (Sigma, St. Louis, MO) in water, and UCF-101 or zVAD.fmk (both from Calbiochem, La Jolla, CA) dissolved in DMSO. Inhibitors were added by replacing culture medium with medium containing inhibitor while control medium included the appropriate solvent (DMSO) or no addition. Morphology and presence of viral nuclear antigens IE1_p72_ and IE2_p86_ were assessed as described above and viral yield was determined from total virus recovered on day 7 from supernatant and sonicated cells [5]. DMSO does not impact CMV death or CMV replication levels at the concentrations used (≤0.1%)[5].

Transfections of BACmid DNA have been described [5]. GFP-positive (GFP+) cells and GFP-positive foci (>2 GFP+ cell) were evaluated by live cell microscopy 2–10 days post transfection. Viral presence was confirmed by immunodection of viral nuclear antigens IE1_p72_ and IE2_p86_ and some experiments utilized a plasmid encoding GFP [5] for detection of transfected cells. Reported results were obtained from multiple DNA preparations.

### Apoptosis and viability assays

Conditions for induced apoptosis of AD*var*ATCC infections and vMIA-dependent survival following transfection of HeLa have been described [2],[64]. Cell numbers were determined following Hoechst stain of surviving cells (HeLas) or following immunodetection of viral nuclear antigens IE1_p72_ and IE2_p86_ (AD*var*ATCC), comparing to untreated controls.

To assess the impact of HtrA2/Omi on HFs, GFP expression plasmid was cotransfected with control DNA (vector) or HtrA2/Omi or HtrA2S306A expression plasmids. By 48 h cells had recovered and were confluent. Images obtained of GFP fluorescence at 24 h intervals between 48–120 h post transfection were evaluated for numbers of GFP+ cells from 12 microscopic fields per day. Mean % survival (±standard deviation) was calculated from numbers of GFP+ cells compared to those at 48 h.

### SLCK labeling

Sulforhodamine 101-leucine chloromethyl ketone, SLCK, (Immunochemistry Technologies, LLC, Bloomington, MN) was suspended in DMSO (Sigma, St. Louis, MO). For labeling, 2.5 µM SLCK was applied in the presence or absence of TLCK to live cultures for 30 minutes prior to fixation and imaging as described above.

## Supporting Information

Figure S1 Towne-BAC cmvPCD occurs after viral release. Examples of single infected cells (A, B) or foci (C, D) remaining intact and live (A, C) or exhibiting fragmentation and death (cmvPCD) (B, D) are shown. Original magnification ×400. (E) Percentages of single infected cells (patterns A+B) and foci (patterns C+D) for ΔUL37x1 and Towne-BAC. (F) Percentages of live, nonfragmented single cells or foci at 72, 96, or 120 h postinfection (hpi) with ΔUL37x1 or Towne-BAC. (G) Percentages of dead single cells or foci at 72, 96, or 120 hpi with ΔUL37x1 or Towne-BAC. A total of 400 infected cells/foci per virus were evaluated at each time for the experiment depicted in panels E–G following infection at MOI 0.0001. The mean±sd is depicted in all figures, except where indicated.
(1.84 MB TIF)

Figure S2 Nuclear and cytoplasmic inclusions indicate similar late cytopathic effects in Towne-BAC and ΔUL37x1 infections. Representative fluorescent images of nuclear and cytoplasmic inclusion proteins ppUL44 (A, C) and ppUL28 (E, I) (red), respectively, in ΔUL37x1 (A–B, E–H) and Towne-BAC (C–D, I–L) infected cells (MOI of 0.001). (GFP = green, Hoechst = blue) Original magnification ×1000.
(6.21 MB TIF)

Figure S3 cmvPCD intermediates do not stain with ethidium homodimer. Representative fluorescent images of GFP (A, E, I, M, Q) (green), ethidium homodimer (B, F, J, N, R, U) (red), Hoechst (C, G, K, O, S, V) (blue), and merged images (D, H, L, P, T, W) in ΔUL37x1 infected intact (A–D) or fragmenting (E–T) cells stained prior to fixation, and in controls fixed with methanol prior to labeling (U, V, W). Original magnification ×1000.
(2.45 MB TIF)

Figure S4 Towne-BAC death intermediates. Representative fluorescent images of Towne-BAC infected cells (MOI of 0.01) showing cmvPCD (A–D) at 168 h postinfection (hpi). Original magnification ×400.
(1.99 MB TIF)

Figure S5 HtrA2/Omi expression following CMV infection. Immunoblot analyses of HtrA2/Omi in lysates of mock-infected HF (M) or HF infected (MOI of 3) with Towne-BAC or ΔUL37x1 for 24, 48, 72, 96, or 120 h. Control cell lysates from HeLa cells transfected with HtrA2/Omi expression plasmid (+) or control plasmid (−). Immunoblot detection of HtrA2, β-actin and IE1 are shown.
(0.50 MB TIF)

Figure S6 Mitochondria are reticular in ΔUL37x1 and punctate in Towne-BAC infections. Representative fluorescent images of mitochondria HSP70 (mtHSP70) (A, E), golgin 97 (I, M), HtrA2/Omi (Q, U), and cytochrome c (Y, c) (blue), and MitoTracker Red stain of mitochondria (B, F, J, N, R, V, Z, d) (red), and GFP fluorescence (C, G, K, O, S, W, a, e) (green) and merged images (D, H, L, P, T, X, b, f) at 72 h postinfection (MOI of 0.001) with ΔUL37x1 (A–D, I–L, Q–T, Y–b) or Towne-BAC (E–H, M–P, U–X, c–f). Original magnification ×1000.
(3.53 MB DOC)

Figure S7 Reticular cytochrome c pattern maintained until after initiation of cmvPCD. Representative fluorescent images of cytochrome c (A, D, G, J, M), GFP fluorescence (B, E, H, K N) and Hoechst (C, F, I, L, O) at 96 h postinfection (MOI of 0.001) in fragmented ΔUL37x1 infected cells. Arrow in B indicates fragments of GFP+ cell. Original magnification ×1000.
(4.19 MB TIF)

Figure S8 HtrA2 overexpression in HeLa cells does not induce death. GFP fluorescence following cotransfection of HF with GFP expression plasmid together with empty vector, HtrA2/Omi or HtrA2S306A expression plasmids at 24, 48, and 72 h posttransfection. Original magnification x40.
(2.56 MB TIF)

Figure S9 HtrA2 reduces plaque formation following a decrease in viability of infected cells evident by 72 h postinfection. Combined numbers of GFP+ cells and foci following cotransfection of Towne-BAC DNA (500 ng) with 800 ng vector or HtrA2/Omi expression plasmid.
(0.16 MB TIF)

Figure S10 vMIA impact on cmvPCD. (A) Total number of ΔUL37x1 plaques on day 10 following infection at MOI 0.0001 of HF, vMIAmut-HF, or vMIA-HF (B) Total viral yield following infection of HF by ΔUL37x1, Towne-BAC, ΔUL37x1R1, or ΔUL37x1R2 for 10 days at MOI 0.0001. (C–H) Mitochondria in ΔUL37x1R1 and ΔUL37x1R2 infected cells at 72 h postinfection. MitoTracker Red stain (C–F) (red), GFP fluorescence (D, G), and the merged images. Original magnification ×1000.
(4.66 MB TIF)

Figure S11 Serine proteases labeled with SLCK and impact of TLCK added as inhibitor. Representative images of fluorescent serine protease substrate SLCK localization (A–C) and GFP fluorescence (D–F) in ΔUL37x1 foci undergoing fragmentation on day 8 postinfection (MOI of 0.001) in the presence of no addition (A, D), 100 µM TLCK (B, E), or 1 mM TLCK (C, F) Original magnification ×200.
(4.49 MB TIF)
