# Supplementary figures and images for: HtrA2/Omi Terminates Cytomegalovirus Infection and Is Controlled by the Viral Mitochondrial Inhibitor of Apoptosis (vMIA)
Source: PLoS Pathog. 2008 May 9;4(5):e1000063. doi: 10.1371/journal.ppat.1000063 (PMC2528007; doi:10.1371/journal.ppat.1000063)

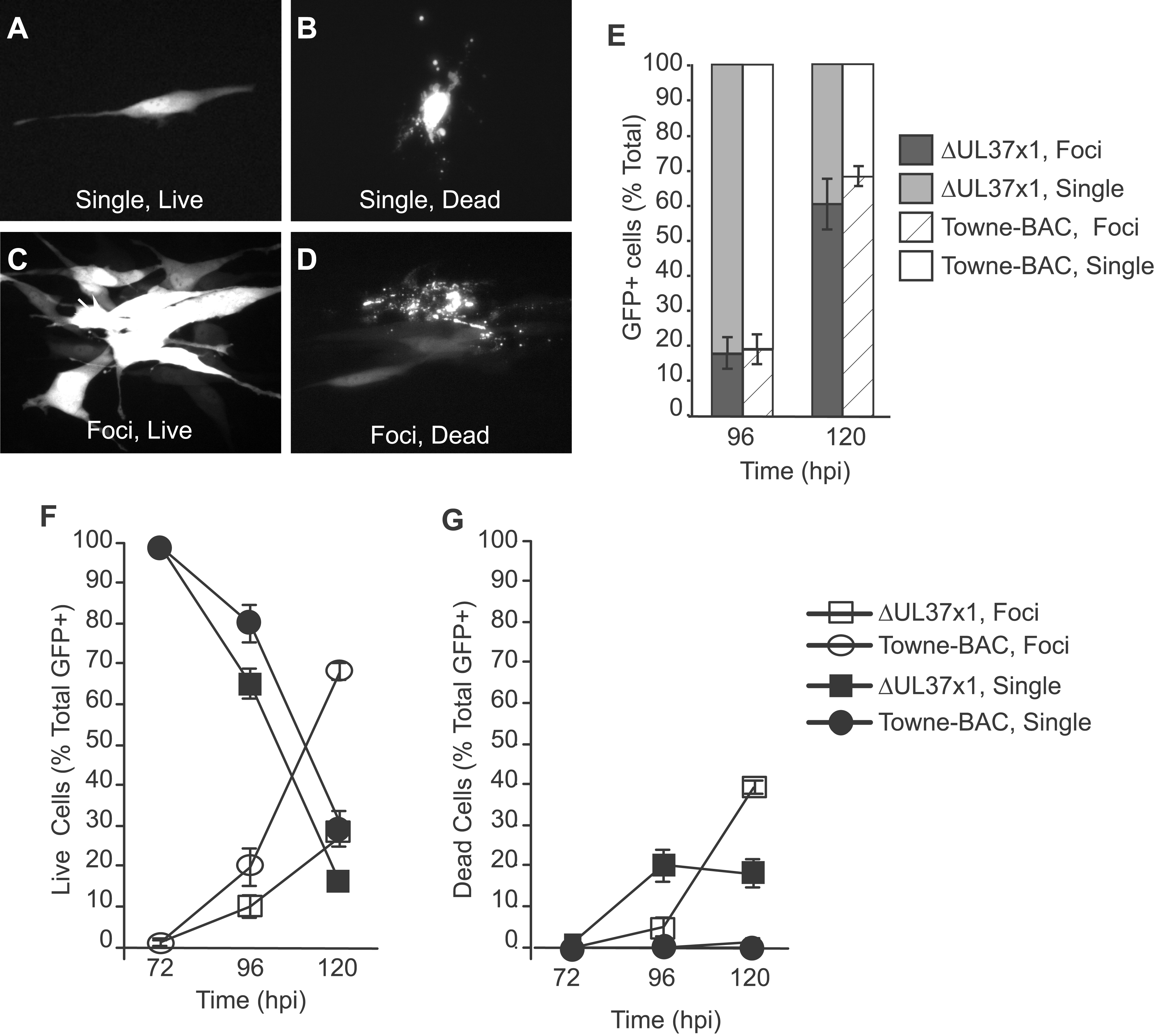

Supplement: Figure S1 — Towne-BAC cmvPCD occurs after viral release. Examples of single infected cells (A, B) or foci (C, D) remaining intact and live (A, C) or exhibiting fragmentation and death (cmvPCD) (B, D) are shown. Original magnification ×400. (E) Percentages of single infected cells (patterns A+B) and foci (patterns C+D) for ΔUL37x1 and Towne-BAC. (F) Percentages of live, nonfragmented single cells or foci at 72, 96, or 120 h postinfection (hpi) with ΔUL37x1 or Towne-BAC. (G) Percentages of dead single cells or foci at 72, 96, or 120 hpi with ΔUL37x1 or Towne-BAC. A total of 400 infected cells/foci per virus were evaluated at each time for the experiment depicted in panels E–G following infection at MOI 0.0001. The mean±sd is depicted in all figures, except where indicated. (1.84 MB TIF) [file ppat.1000063.s001.tif]

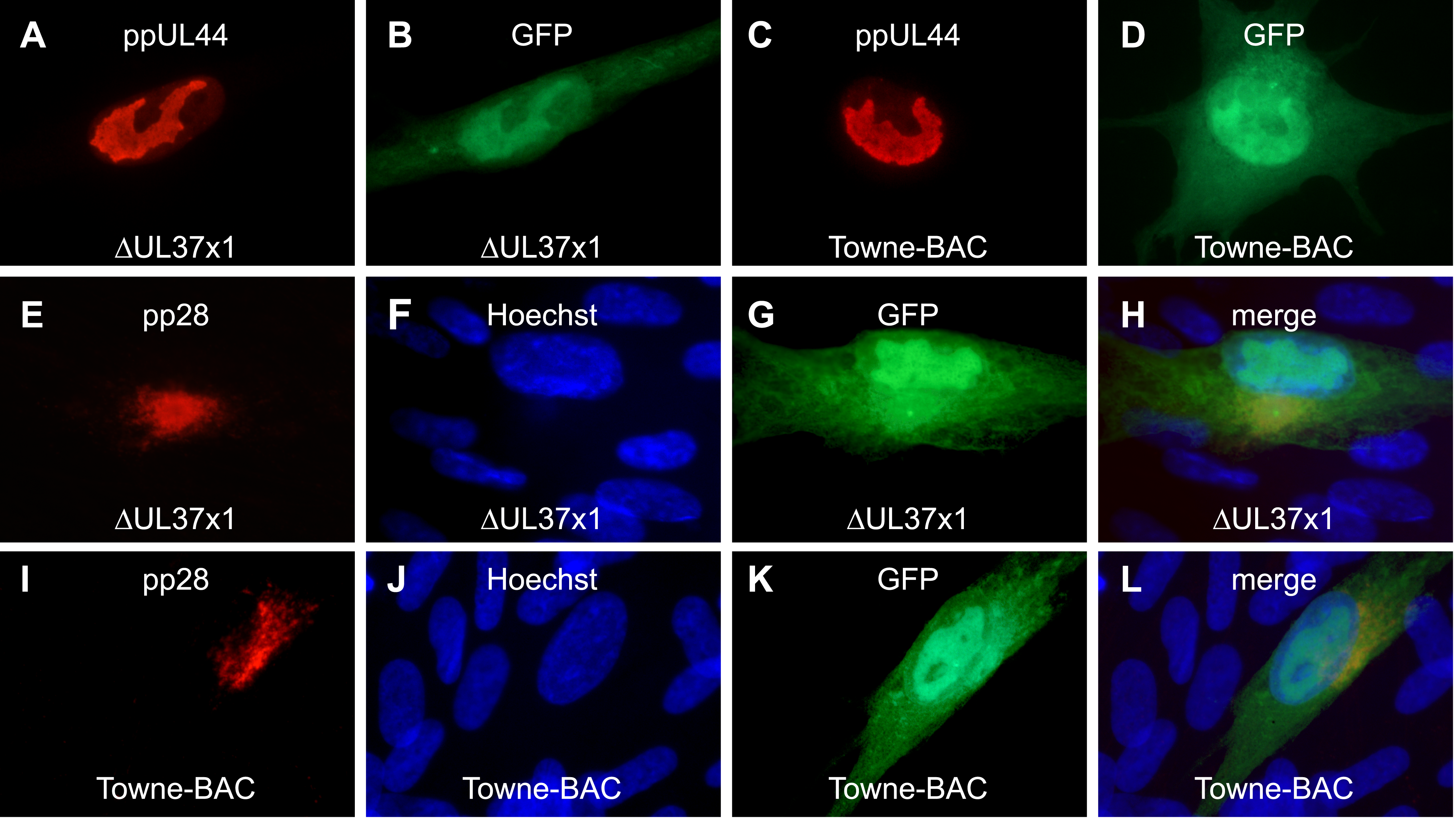

Supplement: Figure S2 — Nuclear and cytoplasmic inclusions indicate similar late cytopathic effects in Towne-BAC and ΔUL37x1 infections. Representative fluorescent images of nuclear and cytoplasmic inclusion proteins ppUL44 (A, C) and ppUL28 (E, I) (red), respectively, in ΔUL37x1 (A–B, E–H) and Towne-BAC (C–D, I–L) infected cells (MOI of 0.001). (GFP = green, Hoechst = blue) Original magnification ×1000. (6.21 MB TIF) [file ppat.1000063.s002.tif]

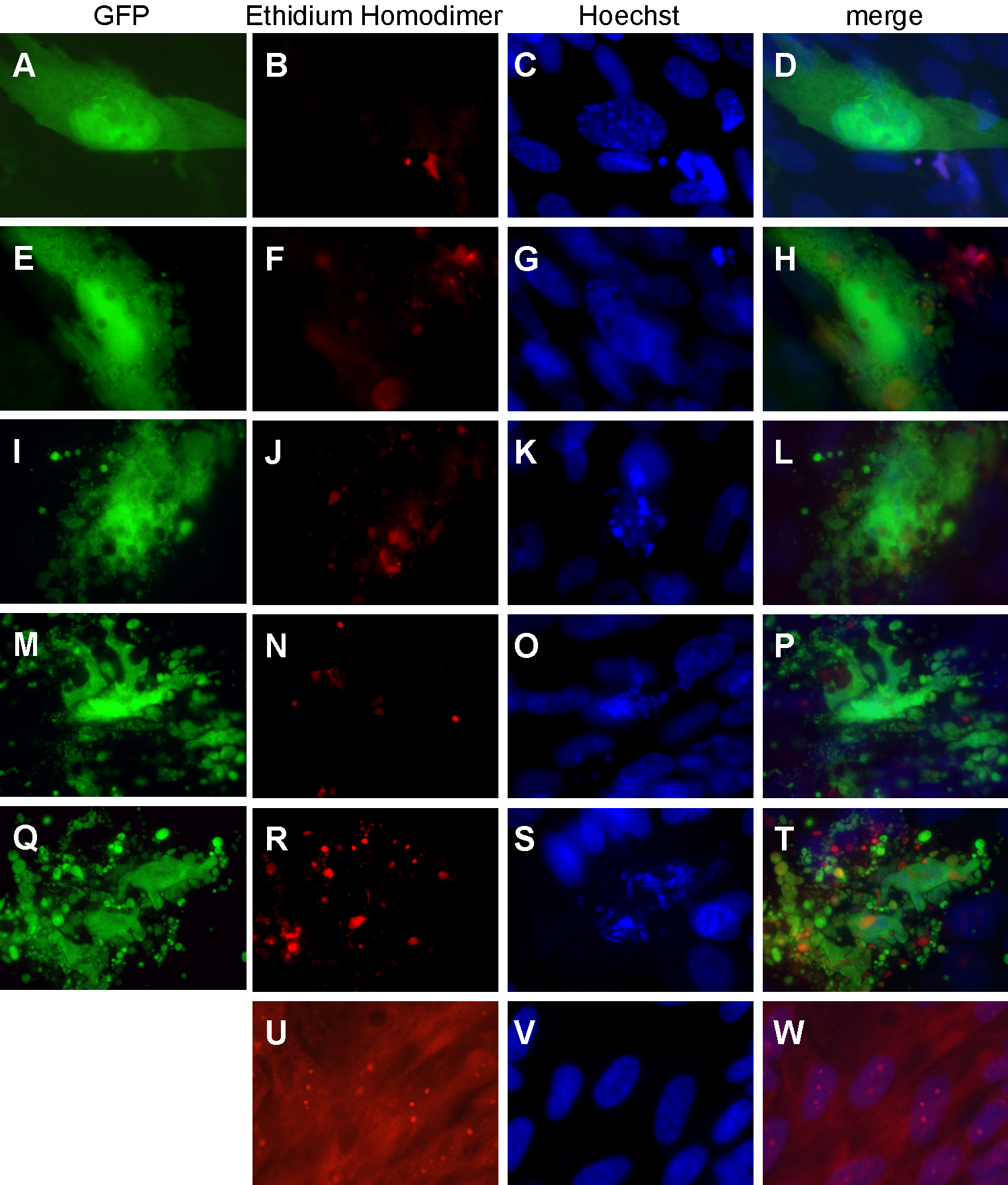

Supplement: Figure S3 — cmvPCD intermediates do not stain with ethidium homodimer. Representative fluorescent images of GFP (A, E, I, M, Q) (green), ethidium homodimer (B, F, J, N, R, U) (red), Hoechst (C, G, K, O, S, V) (blue), and merged images (D, H, L, P, T, W) in ΔUL37x1 infected intact (A–D) or fragmenting (E–T) cells stained prior to fixation, and in controls fixed with methanol prior to labeling (U, V, W). Original magnification ×1000. (2.45 MB TIF) [file ppat.1000063.s003.tif]

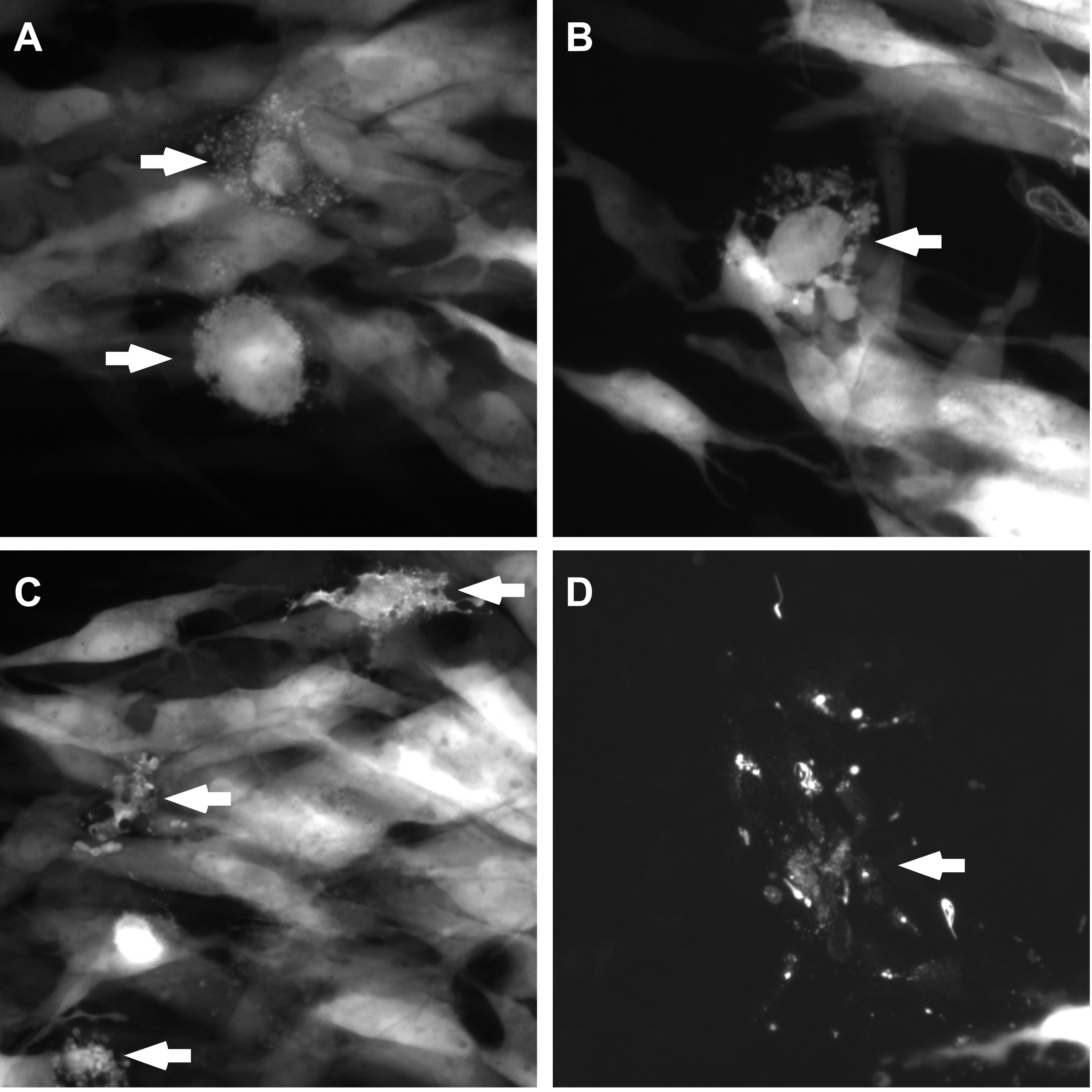

Supplement: Figure S4 — Towne-BAC death intermediates. Representative fluorescent images of Towne-BAC infected cells (MOI of 0.01) showing cmvPCD (A–D) at 168 h postinfection (hpi). Original magnification ×400. (1.99 MB TIF) [file ppat.1000063.s004.tif]

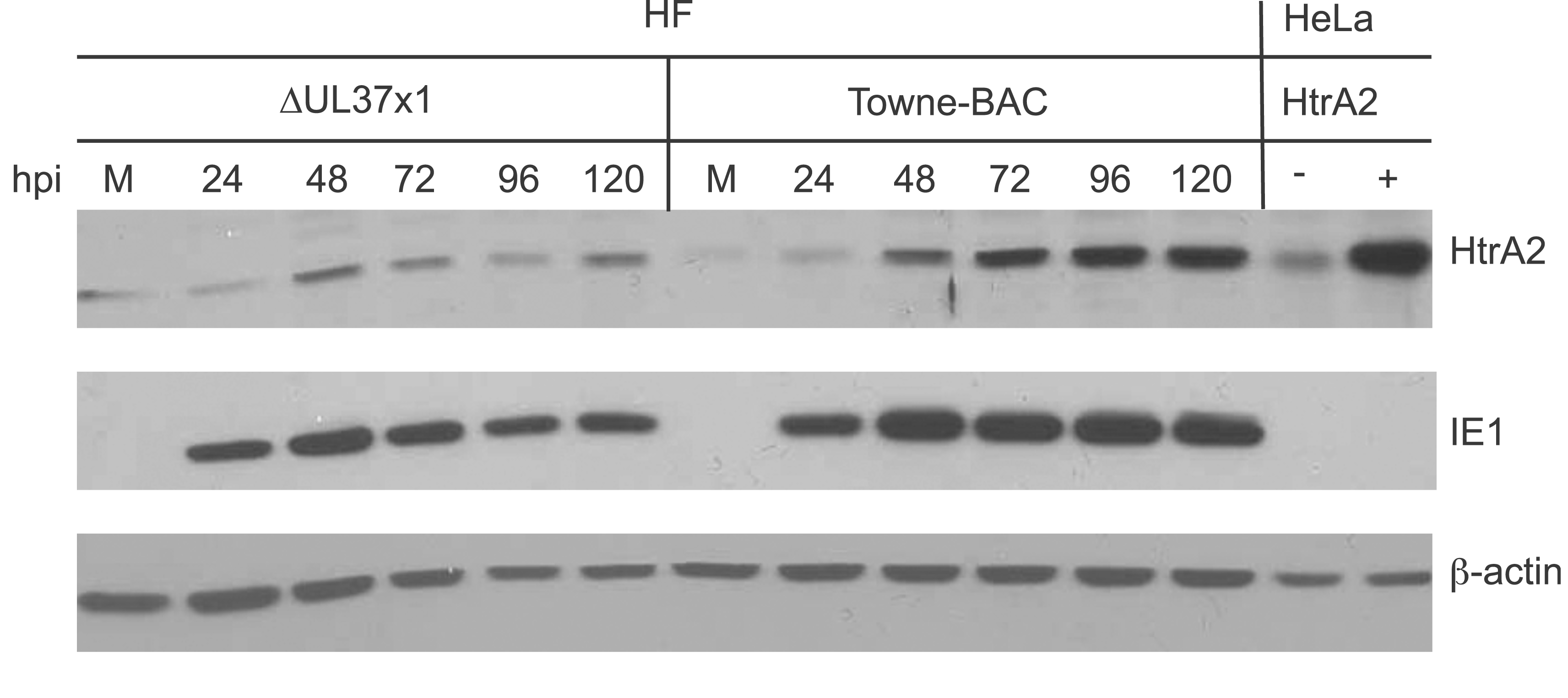

Supplement: Figure S5 — HtrA2/Omi expression following CMV infection. Immunoblot analyses of HtrA2/Omi in lysates of mock-infected HF (M) or HF infected (MOI of 3) with Towne-BAC or ΔUL37x1 for 24, 48, 72, 96, or 120 h. Control cell lysates from HeLa cells transfected with HtrA2/Omi expression plasmid (+) or control plasmid (−). Immunoblot detection of HtrA2, β-actin and IE1 are shown. (0.50 MB TIF) [file ppat.1000063.s005.tif]

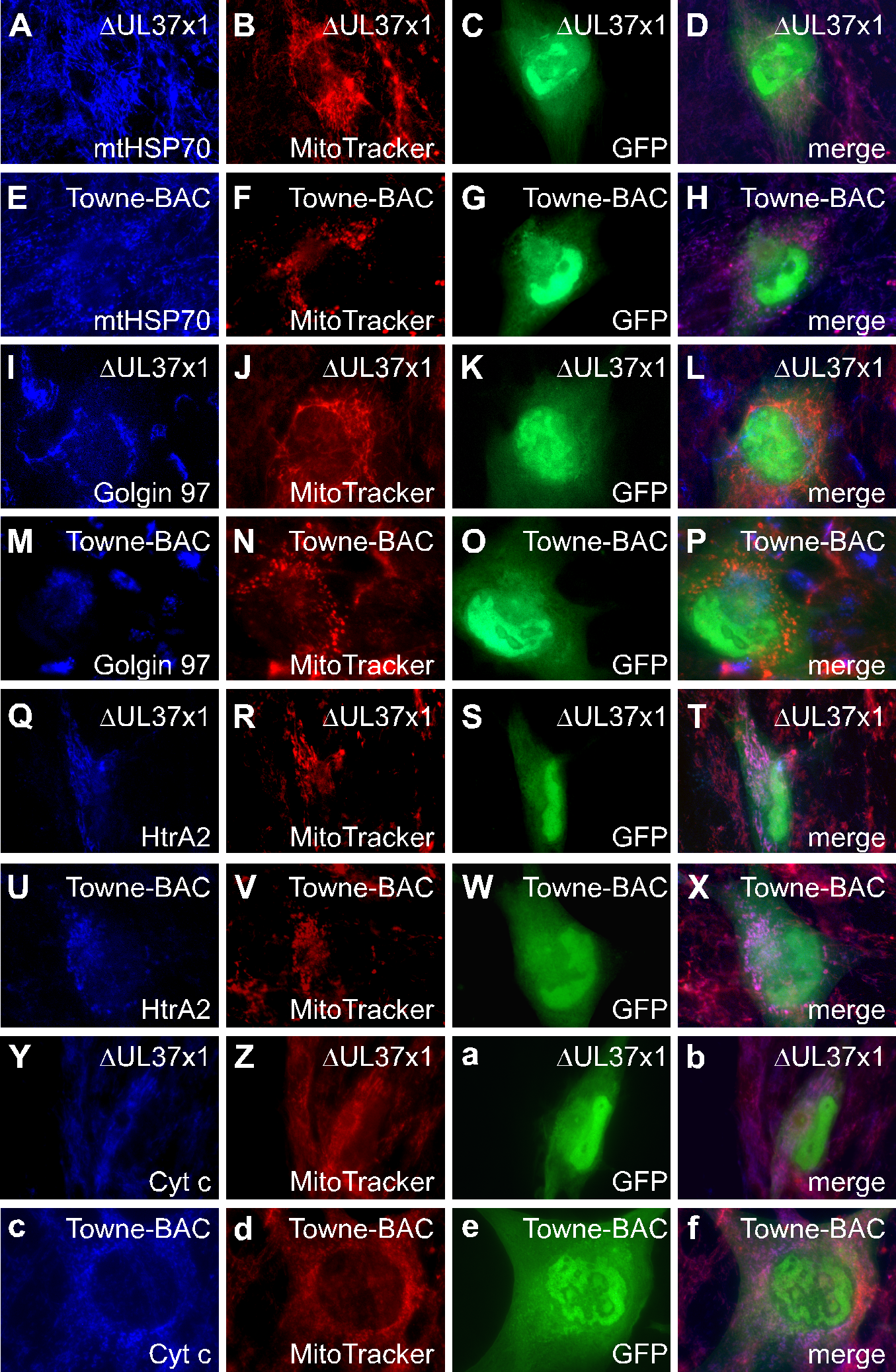

Supplement: Figure S6 — Mitochondria are reticular in ΔUL37x1 and punctate in Towne-BAC infections. Representative fluorescent images of mitochondria HSP70 (mtHSP70) (A, E), golgin 97 (I, M), HtrA2/Omi (Q, U), and cytochrome c (Y, c) (blue), and MitoTracker Red stain of mitochondria (B, F, J, N, R, V, Z, d) (red), and GFP fluorescence (C, G, K, O, S, W, a, e) (green) and merged images (D, H, L, P, T, X, b, f) at 72 h postinfection (MOI of 0.001) with ΔUL37x1 (A–D, I–L, Q–T, Y–b) or Towne-BAC (E–H, M–P, U–X, c–f). Original magnification ×1000. (3.53 MB DOC) [file ppat.1000063.s006.tif]

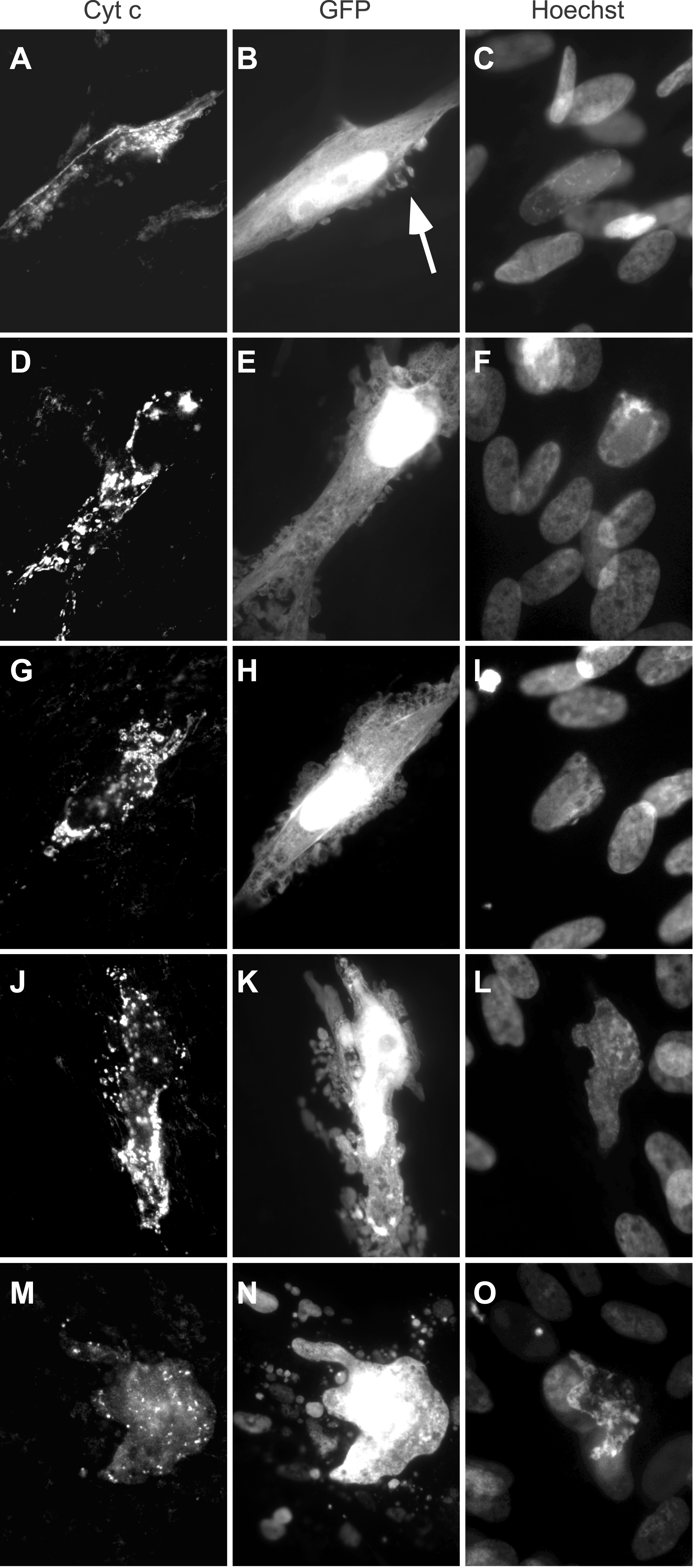

Supplement: Figure S7 — Reticular cytochrome c pattern maintained until after initiation of cmvPCD. Representative fluorescent images of cytochrome c (A, D, G, J, M), GFP fluorescence (B, E, H, K N) and Hoechst (C, F, I, L, O) at 96 h postinfection (MOI of 0.001) in fragmented ΔUL37x1 infected cells. Arrow in B indicates fragments of GFP+ cell. Original magnification ×1000. (4.19 MB TIF) [file ppat.1000063.s007.tif]

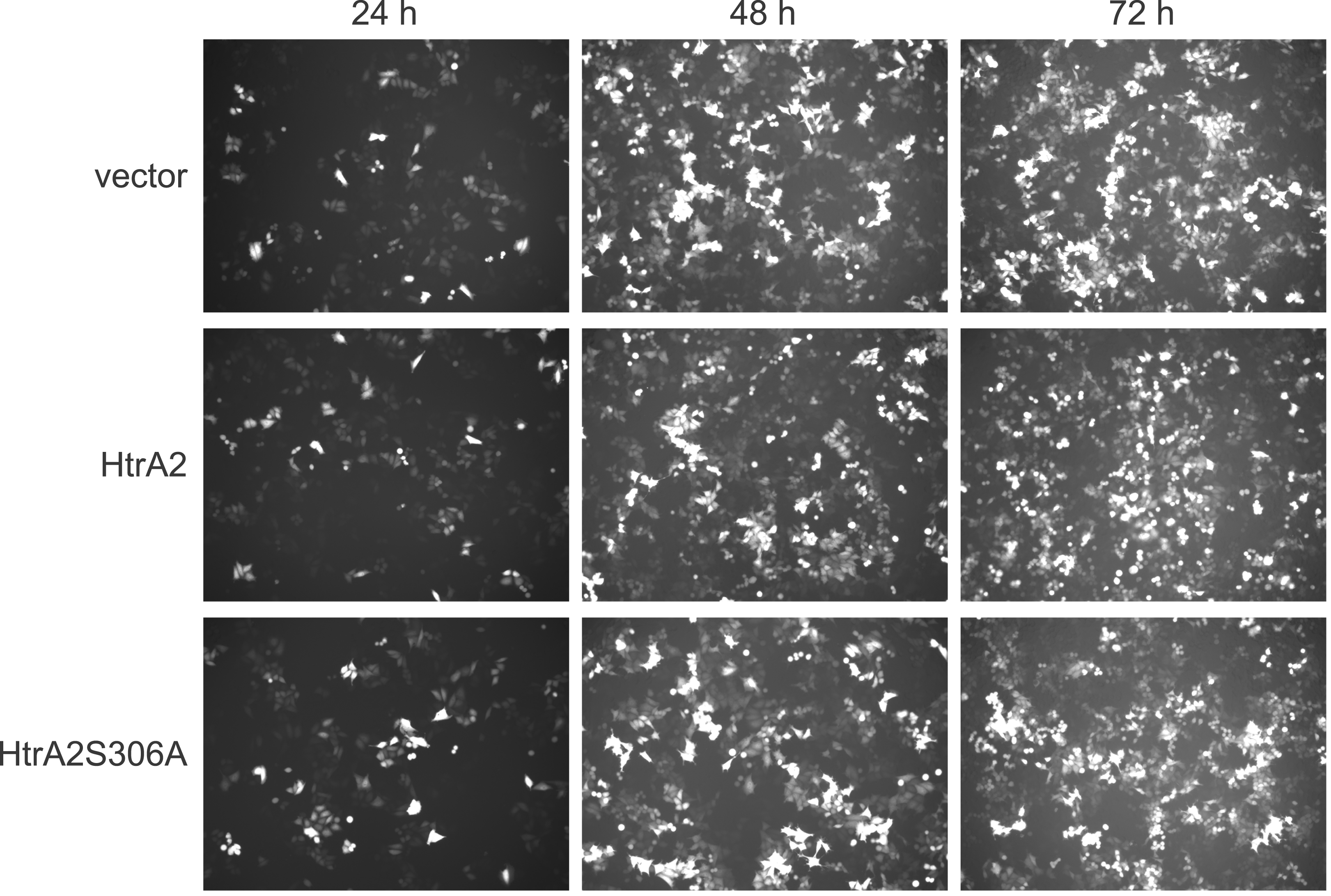

Supplement: Figure S8 — HtrA2 overexpression in HeLa cells does not induce death. GFP fluorescence following cotransfection of HF with GFP expression plasmid together with empty vector, HtrA2/Omi or HtrA2S306A expression plasmids at 24, 48, and 72 h posttransfection. Original magnification x40. (2.56 MB TIF) [file ppat.1000063.s008.tif]

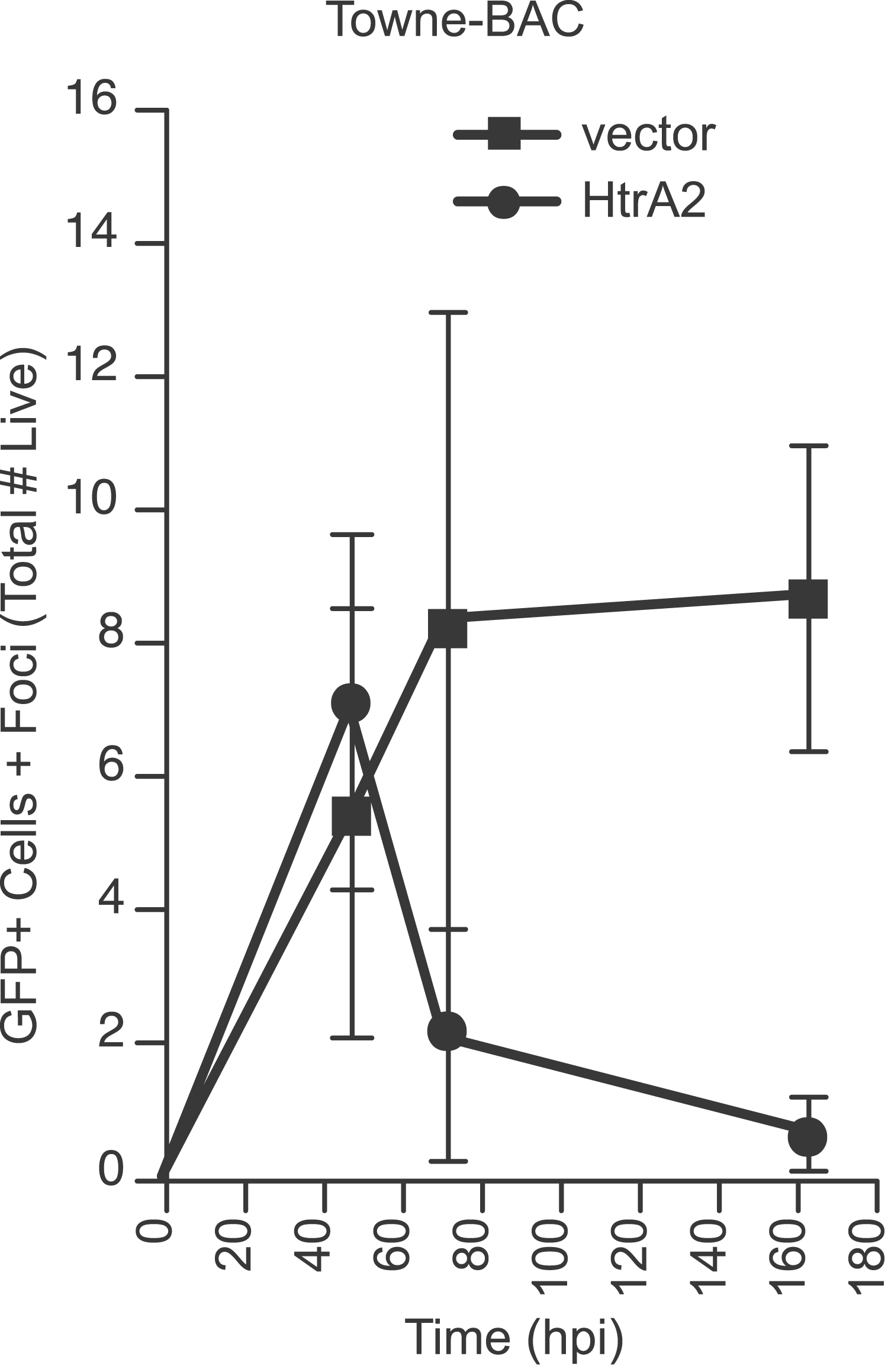

Supplement: Figure S9 — HtrA2 reduces plaque formation following a decrease in viability of infected cells evident by 72 h postinfection. Combined numbers of GFP+ cells and foci following cotransfection of Towne-BAC DNA (500 ng) with 800 ng vector or HtrA2/Omi expression plasmid. (0.16 MB TIF) [file ppat.1000063.s009.tif]

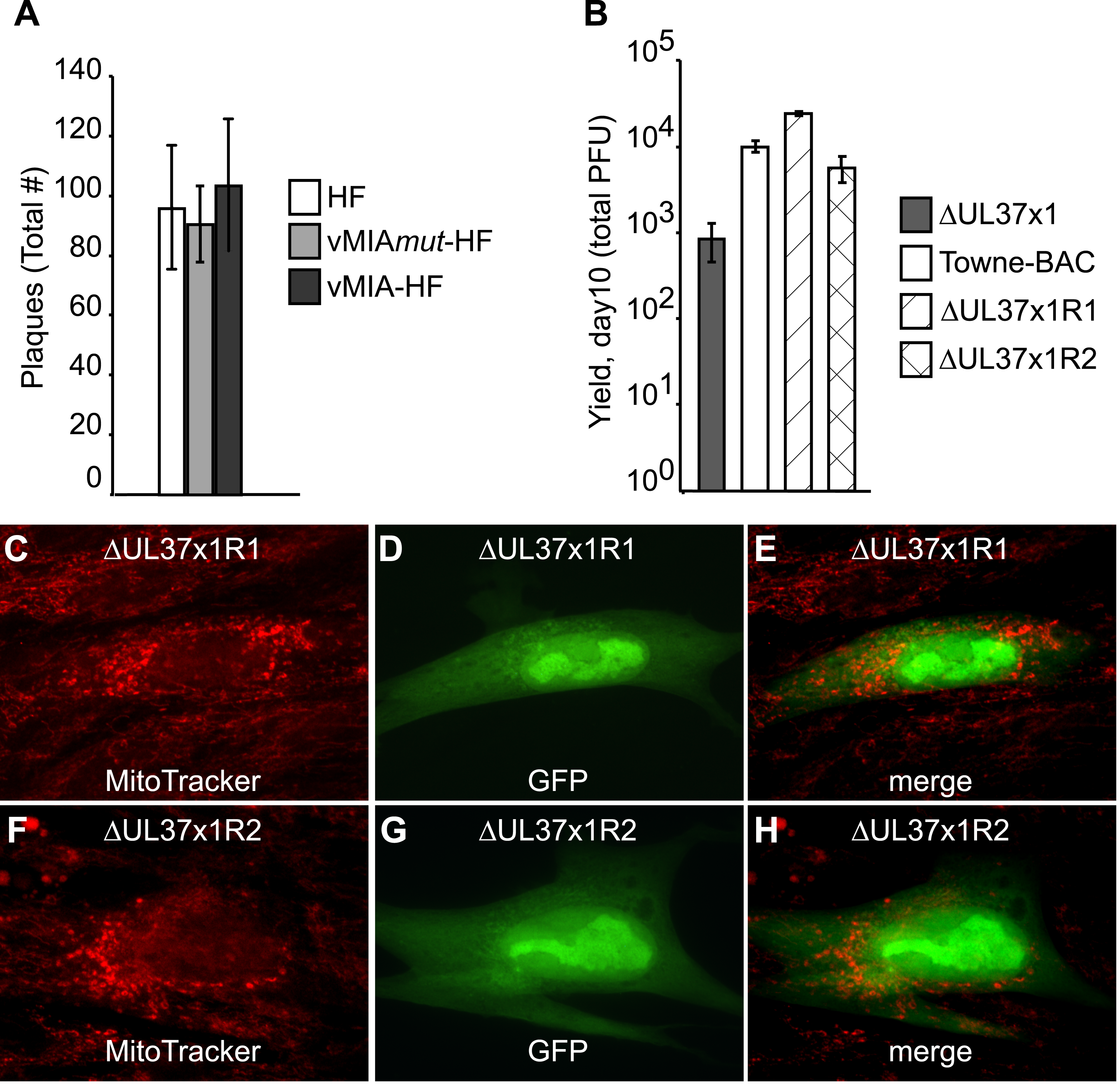

Supplement: Figure S10 — vMIA impact on cmvPCD. (A) Total number of ΔUL37x1 plaques on day 10 following infection at MOI 0.0001 of HF, vMIAmut-HF, or vMIA-HF (B) Total viral yield following infection of HF by ΔUL37x1, Towne-BAC, ΔUL37x1R1, or ΔUL37x1R2 for 10 days at MOI 0.0001. (C–H) Mitochondria in ΔUL37x1R1 and ΔUL37x1R2 infected cells at 72 h postinfection. MitoTracker Red stain (C–F) (red), GFP fluorescence (D, G), and the merged images. Original magnification ×1000. (4.66 MB TIF) [file ppat.1000063.s010.tif]

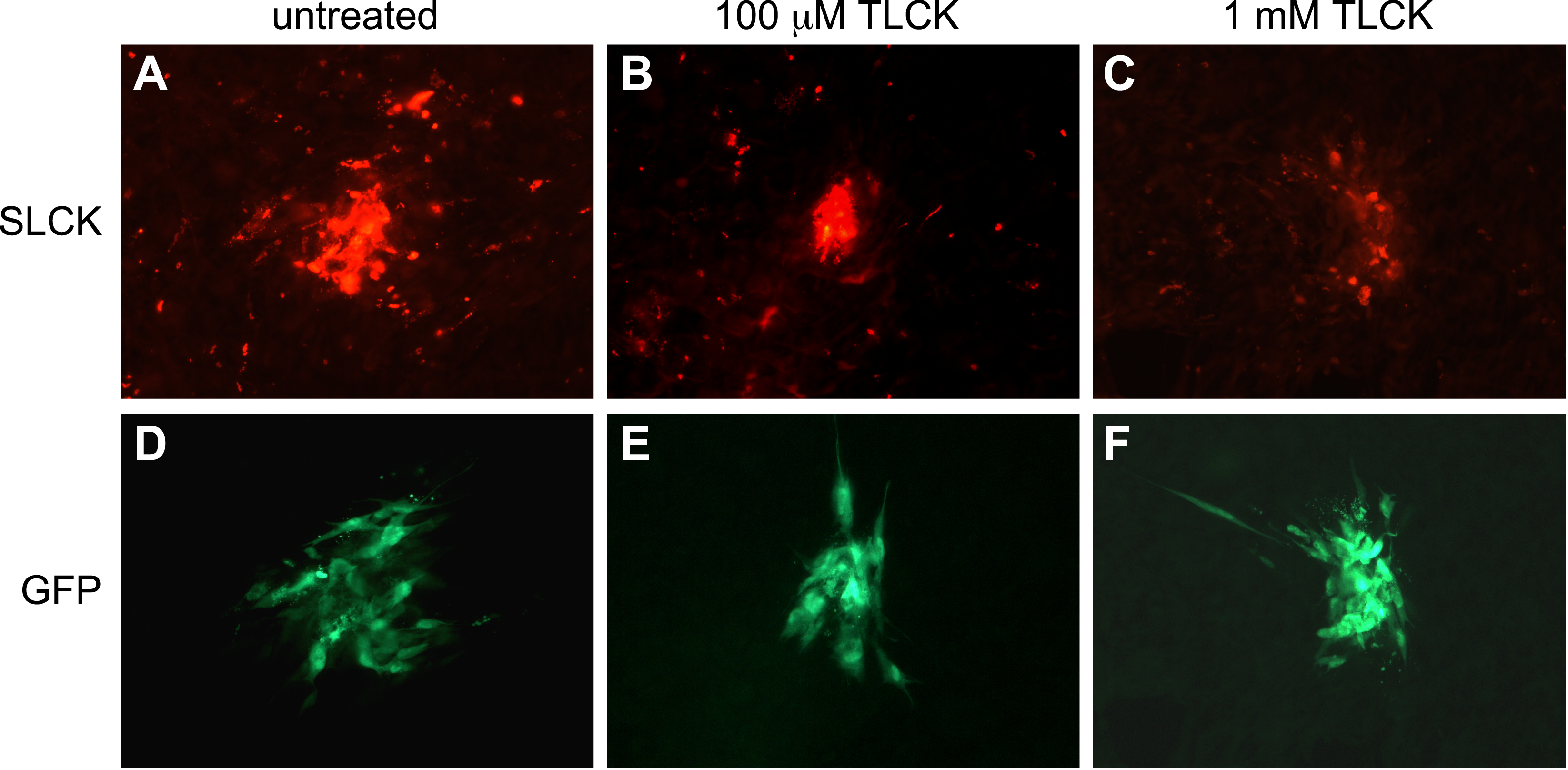

Supplement: Figure S11 — Serine proteases labeled with SLCK and impact of TLCK added as inhibitor. Representative images of fluorescent serine protease substrate SLCK localization (A–C) and GFP fluorescence (D–F) in ΔUL37x1 foci undergoing fragmentation on day 8 postinfection (MOI of 0.001) in the presence of no addition (A, D), 100 µM TLCK (B, E), or 1 mM TLCK (C, F) Original magnification ×200. (4.49 MB TIF) [file ppat.1000063.s011.tif]
